# Exploring Marine Biomineralization on the Al–Mg Alloy as a Natural Process for In Situ LDH Growth to Improve Corrosion Resistance

**DOI:** 10.1021/acsami.4c17532

**Published:** 2025-01-30

**Authors:** Maria João F. Marques, Dimitri Mercier, Antoine Seyeux, Sandrine Zanna, Christophe Tenailleau, Benjamin Duployer, Marc Jeannin, Philippe Marcus, Régine Basséguy

**Affiliations:** † Laboratoire de Génie Chimique, CNRS, INPT, UPS, Université de Toulouse, Toulouse 31432, France; ‡ Laboratório de Materiais e Revestimentos, Laboratório Nacional de Energia e Geologia (LNEG), Lisboa 1649-038, Portugal; § Institut de Recherche de Chimie Paris, Research Group Physical Chemistry of Surfaces, Chimie ParisTech-CNRS, 27051 PSL Research University, Paris 75005, France; ∥ Centre Interuniversitaire de Recherche et d’Ingénierie des Matériaux, CNRS, UPS, Université de Toulouse 3, Toulouse 31062, France; ⊥ 531131 Laboratoire des Sciences de ÍIngénieur pour ÍEnvironnement, CNRS, Université de La Rochelle, La Rochelle 17042, France

**Keywords:** aluminum alloy, in situ growth of LDH, biomineralization, marine corrosion inhibition, marine photosynthetic fouling, sulfated polysaccharides

## Abstract

This study provides a detailed characterization of the AA5083 aluminum alloy, surface, and interface over 6 months of immersion in seawater, employing techniques such as SEM/EDX, GIXRD, μ-Raman and XPS. The purpose was to evaluate the evolution of the biomineralization process that occurs on the Al–Mg alloy. By investigating the specific conditions that favor the in situ growth of layered double hydroxide (LDH) during seawater immersion as a result of biomineralization, this research provides insights into marine biomineralization, highlighting its potential as an innovative and sustainable strategy for corrosion protection.

## Graphical abstract

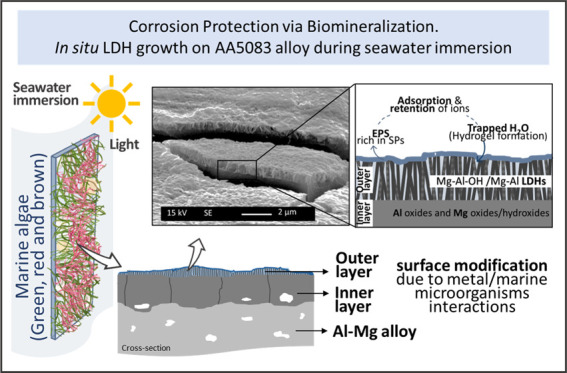

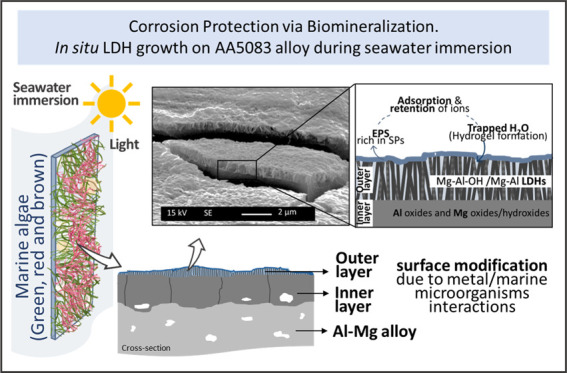

## 1 Introduction

Aluminum–magnesium (Al–Mg) alloys are widely utilized in marine, automotive, and aerospace applications due to their excellent mechanical properties, low density, and inherent corrosion resistance. Despite these advantages, Al–Mg alloys are vulnerable to localized corrosion, particularly in marine environments where chloride ions and other corrosive species are present.^1–7^ Traditional methods for improving the corrosion resistance of Al–Mg alloys often involve complex and costly processes associated with significant energy consumption as well as raising environmental concerns. As a consequence, there is an increasing demand for sustainable and environmentally friendly corrosion protection strategies.^8–11^ In the last decades, bioinspired and environmentally friendly strategies have garnered attention as alternatives for anticorrosive protection. Among these, marine biomineralization—a natural process by which marine organisms form mineralized structures—has been proven to enable the formation of protective layers on metallic surfaces, offering a promising pathway for developing new bioinspired corrosion solutions.^12–16^ Even though the studies in this field have been focused essentially on steel,^17–28^ in recent years, the research on aluminum alloys has been increasing.^29–32^ Nevertheless, the majority of the published research continues to be carried out in a laboratory environment, considering the influence of only one species of bacteria or microorganisms in the marine biomineralization process, with very few studies being performed under field conditions.^33,34^

Recently, with the aim of progressing in the understanding of the characteristics and properties of the marine biomineralization layer formed on the surface of Al–Mg alloy, the authors of the present article published a study that focused on the chemical and structural characterization of the surface modifications that occurred on the AA5083 alloy during 2 months of immersion in natural seawater.^35^ It was found that the corrosion process was different according to the solar radiation, which induced a distinct development of marine fouling on the Al–Mg surfaces. In the dark (shade exposure), where the presence of hard fouling was mainly present, a single thick layer rich in Al oxides/hydroxides was formed. This layer did not show a barrier effect to Cl^–^ and pitting of the Al–Mg alloy was observed after two months of seawater immersion. In the case of the light side (direct exposure to sunlight), where the presence of soft fouling was mainly observed, a dual layer structure was formed in which a hydrated Mg-rich outer layer with extracellular polymeric substances (EPS) on the top was present, and no localized corrosion was detected. Additionally, a biotic versus abiotic approach enabled verification of the inhibitory effect induced by marine biological activity on the corrosion of the Al–Mg alloy. Although the presence of a Mg-rich outer layer was observed for both cases (biotic/light side and abiotic conditions), its protective effect has been shown to be conditioned by the type of mineralization phenomenon with or without biological activity. In the biotic case, the presence of marine photosynthetic organisms (algae and micro algae) proved to be responsible for the production of specific EPS and the hydration of the Mg-rich outer layer, which impacted the corrosion resistance properties of the layer by reducing Cl^–^ penetration. The Al–Mg surface immersed in abiotic conditions, having an outer layer thicker and more homogeneously distributed than that observed for the biotic conditions, did not show a better barrier effect on Cl^–^ penetration.

The present work is the follow-up of these results, in which it was considered relevant to assess the evolution of the biomineralization process on the Al–Mg alloy surface over a longer period of immersion, with the aim of confirming the prevalence of no pitting attack of the substrate, simultaneously following the behavior of the biomineralized layer and understanding its corrosion-inhibiting effect.

For this purpose, a detailed characterization of the surface and interface of the Al–Mg alloy was performed after the first 15 days of immersion in seawater and until 6 months of exposure, involving different analysis techniques, such as scanning electron microscopy (SEM) with energy dispersive X-ray analysis (EDX), grazing incidence X-ray diffraction (GIXRD), μ-Raman spectroscopy (μ-RS), and X-ray photoelectron spectroscopy (XPS).

## 2 Materials and Methods

### 2.1 Samples Preparation

The AA5083-H111 aluminum alloy (Al–Mg) was supplied by Comptoir Général des Métaux, Cugnaux, France. The elemental chemical composition is given in Table 1. The hot-rolled plates acquired, 1 mm-thick, were cut into test samples (10 × 20 cm^2^). The material was used as received in an initial rough grinding state, with no additional surface preparation given. Before immersion, surface samples were carefully cleaned with water and alcohol and then dried with air.

**Table 1:** Chemical Composition of the Al–Mg Alloy Samples (Extracted from Supplier Certificate)

	Chemical composition (wt %)								
**AA5083-H111**	**Mg**	**Mn**	**Fe**	**Si**	**Cr**	**Cu**	**Zn**	**Ti**	**Al**
	4.35	0.50	0.22	0.11	0.067	0.065	0.018	0.016	balance
min.	4.00	0.40	–	–	0.050	–	–	–	
max.	4.90	1.00	0.40	0.40	0.25	0.10	0.25	0.15	

### 2.2 In Situ Marine Immersion Test

The marine immersion test was carried out on the platform available at the CNR-IAS Genoa Experimental Marine Station (GEMS) (Figure S1), located within the Port of Genoa, Genoa, Italy.^36^

Two immersion campaigns were carried out in natural Mediterranean Seawater, C1 and C2. In both cases, the Al–Mg alloy samples were immersed vertically with two sides of exposure: light (direct sunlight) and dark side (in the shade).

The C1 campaign began on September 1 and ended on November 2, 2020, in which a set of 24 samples of aluminum alloy were immersed, and gradually recovered after 15 days, 1 and 2 months. During the immersion test, the following seawater parameters were daily measured: temperature (average = 22.1 ± 2.4 °C; min = 17.8 °C; max = 26.5 °C), conductivity (average = 54.0 ± 4 mS/cm; min = 45.8 mS/cm; max = 58.1 mS/cm), salinity (average: 37.5 ± 0.9%; min = 35%; max = 38.3%), and pH (average: 8.1 ± 0.1; min = 8.02; max = 8.21).^37^

For the C2 campaign, a set of 8 samples of the AA5083 alloy were immersed in natural Mediterranean seawater for 6 months (between May and November 2021). The following seawater parameters were measured during immersion: temperature (average = 22.6 ± 2.9 °C; min = 16.8 °C; max = 27.1 °C), conductivity (average = 56.7 ± 3.4 mS/cm; min = 49.1 mS/cm; max = 61.5 mS/cm), salinity (average: 37.8 ± 0.6%; min = 36.2%; max= 38.4%), and pH (average: 8.2 ± 0.1; min = 7.9; max = 8.6).^38^

### 2.3 Surface and Interface Characterization after Immersion Test

At the end of each time of immersion, 15 days, 1, 2, and 6 months, the AA5083 samples were rinsed with flowing deionized water and dried under atmospheric conditions before proceeding with characterization.

Since the objective of this study was not to characterize the biofilm formed on the surface of the Al–Mg alloy during immersion but rather to assess the surface transformation as a result of microbiological activity, no chemical fixation process was performed. The entire characterization process of the immersed aluminum alloy samples (light and dark side of exposure) was carried out after a surface cleaning procedure (removal of biofouling). The only exception was the observation by digital microscopy, in which the surfaces before cleaning (with biofouling) were also examined.

The procedure for cleaning the biofouling from the AA5083 samples was carried out as follows: the sample surfaces were cleaned manually using nonmetallic spatulas and under a constant flow of tap water in order to facilitate the removal of the biofouling, avoiding performing excessive force. In the end, the AA5053 samples were then rinsed with deionized water and air-dried under atmospheric conditions before surface characterization.

#### 2.3.1 Digital Microscopy

The AA5083 samples of the C1 and C2 campaigns were observed with a DVM6 Leica digital microscope before and after the cleaning procedure of the biofouling present on the surfaces.

#### 2.3.2 Scanning Electron Microscopy (SEM) with Energy Dispersive X-ray Analysis (EDX)

The aluminum alloy samples immersed for 15 days, 1, 2, and 6 months were characterized, both surface and cross-section, by SEM/EDX. The SEM observations were performed using a JSM-7100F JEOL and a Philips FEG-SEM mode XL30 microscope coupled with a Pathfinder Thermo Fisher Scientific energy dispersive X-ray spectrometer (EDX) for elementary chemical analysis.

The cross-sectional samples were prepared using a cold mounting epoxy resin. Before metallographic preparation, the surface of some samples was previously coated with a fine deposit of platinum in order to improve the observation process. To reduce charging effects, surface and cross section samples were coated with a sputtered layer of gold (Au).

SEM characterization also included an observation after a bending procedure of the samples under study in order to obtain additional information.

#### 2.3.3 Grazing Incidence X-ray Diffraction (GIXRD)

A GIXRD analysis on the 6 month-immersed Al–Mg samples (light and dark side of exposure) was conducted using a Bruker D8 advance, with Cu Kα radiation (λ = 1.5406 Å) operating at 40 kV and 40 mA, with a scan step of 0.02° and 2 s of acquisition per counting step, i.e., 2 h of acquisition per scan, in the 2θ range from 10 to 80° (an angle of incidence of 1°). A LYNXEYE XE-T detector was used in the 0D mode (single point). The peaks of the diffractograms were identified with recourse to the JCPDS-ICDD database (2021).

#### 2.3.4 μ-Raman Spectroscopy (μ-RS)

μ-RS was performed with a Jobin Yvon High-Resolution Raman spectrometer (LabRAM HR-Evo) equipped with a microscope (Olympus BX 41) and a Peltier-based cooled charge-coupled device detector (CCD). The analyzed zones (a diameter of 2 μm) were observed through a long working distance ×50 objective. Spectra were recorded with the acquisition LabSpec software at RT with a resolution of 0.4 cm^–1^. Excitation was provided by a laser diode at 532 nm. Its power varied from 0.06 (1% of the maximal power) to 6 mW (100% of the maximal power). Samples were directly placed on microscope glass under the microscope. Each sample was characterized via at least 20–30 Raman spectra to detect the heterogeneities of oxide and mineral particles.

#### 2.3.5 X-ray Photoelectron Spectroscopy (XPS)

For the XPS analysis of the surface chemical composition, a ThermoElectron ESCALAB 250 Xi spectrometer was used with a monochromatized Al Kα radiation (1486.6 eV). The analyzer pass energy was 100 eV for survey spectra and 20 eV for high resolution spectra. The following core level spectra were recorded: Al 2p, Mg 2p, S 2p, P 2p, Cl 2p, O 1s, C 1s, N 1s, Ca 2p, Na 1s, and Si 2p. Curve fitting of the spectra was performed with the Thermo Electron software “Avantage.”

##### 2.3.5.1 XPS In-Depth Analysis of Surface Layers after Immersion

The Al–Mg in-depth profiling, after 15 days, 1, 2, and 6 months of immersion, was carried out combining a sequence of Argon ions etching cycles with XPS analysis. Using a 1 kV ion beam, three etching cycles of 200 s each (around 0.2 nm.s^–1^) were performed, reaching a final depth of approximately 100 nm, after a total of 600 s of etching.

##### 2.3.5.2 XPS Characterization of Organic Compounds Present on Al–Mg Surfaces

Calculations of protein, polysaccharide, and oxygenated species molar ratios were performed from data extracted from C 1s and N 1s XPS core levels.^39^ The C 1s core level is decomposed into four components attributed to C1 (C–C, C–H bonds at 285 eV), C2 (C–O, C–N at 286.6 eV), and C3 (O–C–O; *N*=C–O at 288.2 eV), with all three associated with the organic matter, and C4 (carbonate species) at 289.8 eV. The N 1s spectrum is attributed to amide and amine at 400.1 eV.

C1 is the molar quantity of carbon bound to carbon and hydrogen. It corresponds to hydrocarbons in lipids and side chains of polysaccharides and proteins or carbonaceous contamination.

C2 corresponds to the molar quantity of carbon in alcohol (found in polysaccharides including amino sugars and uronic acids) and in amine (found in proteins, amino acids, and amino sugars) or amide/peptide bond (found in proteins).

C3 is the sum of carbon in *N*=C–O (peptidic bond) and O–C–O bonds in polysaccharides. C3 corresponds to carbon in polysaccharides including amino sugars and uronic acids or an amide/peptide bond (found in proteins).

N corresponds to the molar quantity of proteins.

Csac corresponds to the molar quantity of carbon in polysaccharides. Csac was calculated by subtracting C–N bonds in proteins to the C2 component (Csac = C2 – N).

- The molar quantity of carbon involved in the peptidic bond in proteins is Cpro = C3 – (1/5) Csac. It is known that the atomic ratio C–C–O/O–C–O is 5 in polysaccharides.^40,41^

Cox is associated with other oxygenated species than proteins and polysaccharides, i.e., lipids, uronic acids, Cox = C2 + C3 – Cpro – Csac.

## 3 Results and Discussion

The visual inspection globally performed on AA5083 samples immersed for 15 days and 1, 2, and 6 months revealed reproducible behavior for the different replicates tested.

Figure 1 shows the evolution of biofouling on the Al–Mg surface with immersion time and the different behavior between the light and dark sides of exposure from the first days of immersion. The light side shows a progressive development of soft fouling on the Al–Mg surface (example of noncalcareous fouling organisms, such as algae, hydroids, tunicates, etc.), contrary to the dark side, where harder fouling is observed (example of calcareous fouling organisms, including barnacles, tubeworms, mussels, etc.).

**Figure 1:**
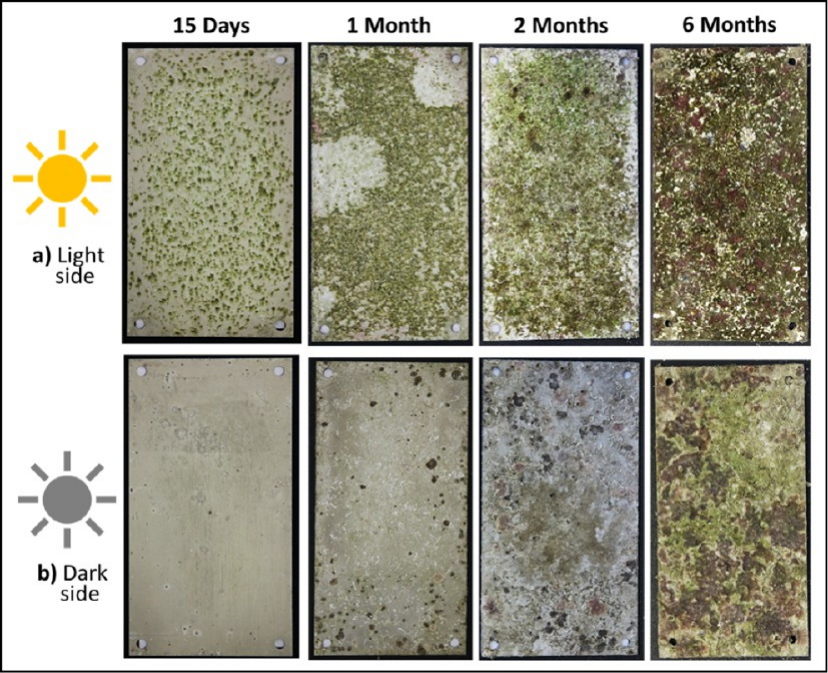
Digital photographs of Al–Mg samples (10 cm × 20 cm) after 15 days and 1, 2, and 6 months of immersion, light (a) and dark (b) sides of exposure, and before the biofouling cleaning procedure.

After the cleaning of the samples following the procedure detailed previously (Figure 2), it was observed that the distinct fouling community on the Al–Mg samples at the end of each immersion time, light and dark side, has led to differences in the appearance of the surface below the fouling. Remains of limestone structures are evidenced on the dark side especially after 2 months of exposure, contrary to the light side.

**Figure 2:**
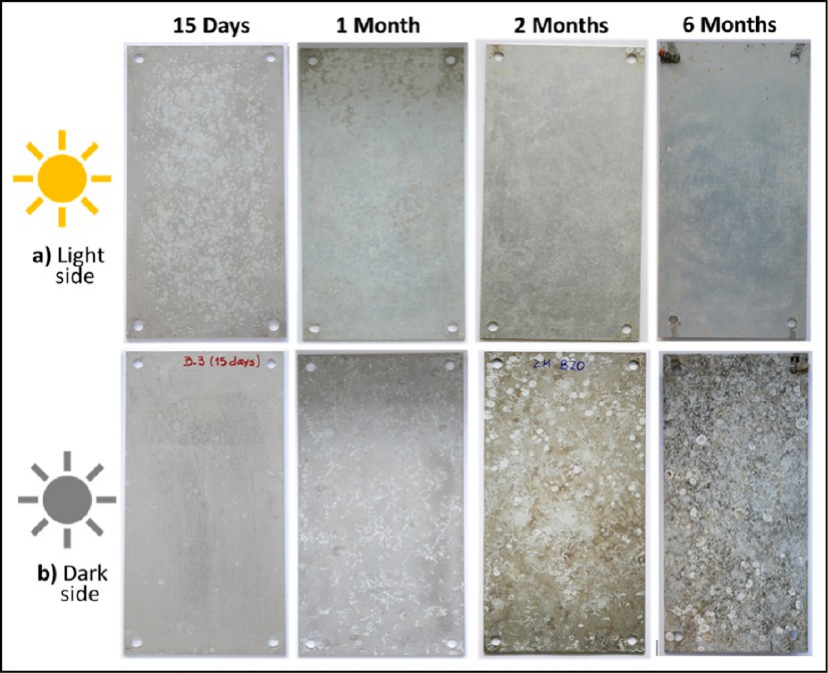
Digital photographs of Al–Mg samples (10 cm × 20 cm) after 15 days and 1, 2, and 6 months of immersion, light (a) and dark (b) sides of exposure, and after the biofouling cleaning procedure.

### 3.1 Digital Microscopy

Figures 3 and 4 show the digital microscope observations of the AA5083 samples after immersion for both sides of exposure before and after removing the biofouling from the surface. The observations confirm the different typologies of biofouling on the aluminum alloy surfaces during the immersion time according to the exposure side, as well as its impact on the surface modifications.

**Figure 3:**
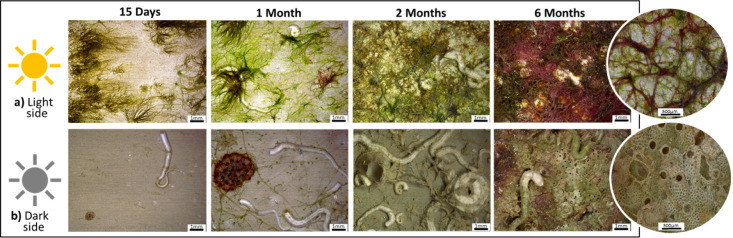
Digital microscope observations of Al–Mg surfaces after 15 days and 1, 2, and 6 months of immersion, light (a) and dark (b) sides of exposure, and before the biofouling cleaning procedure. The images shown in circular format correspond to higher magnifications of the Al–Mg surfaces after 6 months of immersion, with light and dark exposure, respectively.

**Figure 4:**
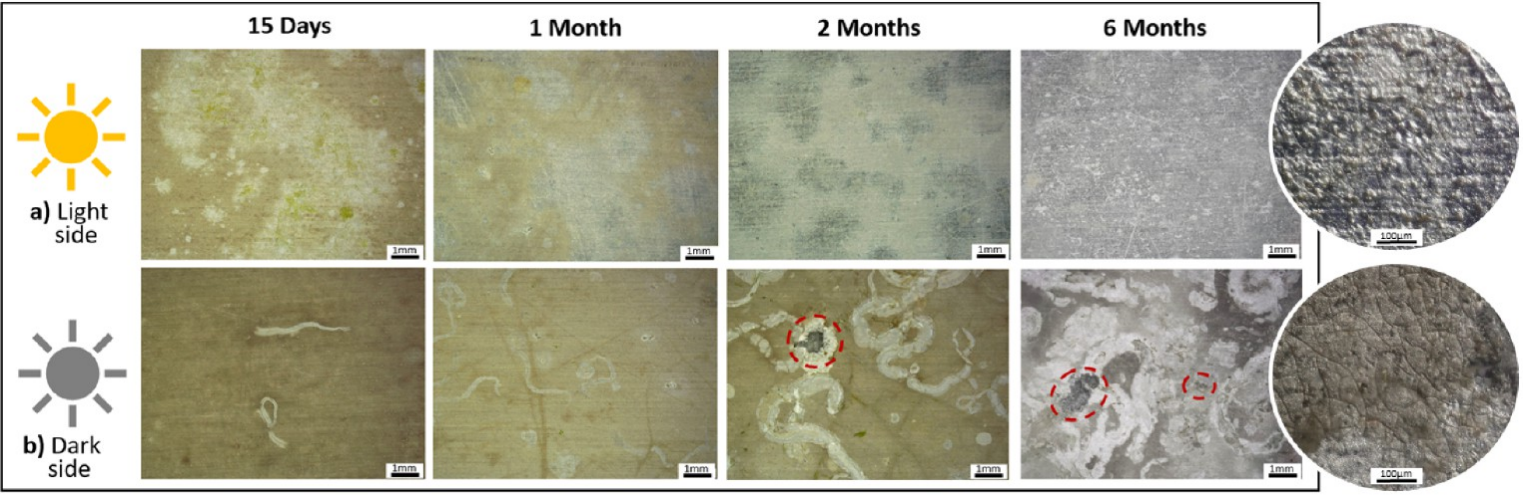
Digital microscope observations of Al–Mg surfaces after 15 days and 1, 2, and 6 months of immersion, light (a) and dark (b) sides of exposure, and after the biofouling cleaning procedure. The images shown in circular format correspond to higher magnifications of the Al–Mg surfaces after 6 months of immersion, with light and dark exposure, respectively.

For the light side (Figure 3), the development of soft fouling on the Al–Mg surface is confirmed after the first 15 days of immersion. The presence of different species of algae (green, brown, and red) and just a few calcareous tubes made by tube worms is progressively observed on the surface as the immersion time evolves. At the end of 6 months of immersion, a significant presence of red algae can be observed on the alloy surface. In contrast, significantly fewer algae are observed on the Al–Mg surfaces on the dark side with predominantly harder fouling organisms such as calcareous deposits, including tube worms and barnacles, being visible (Figure 3b). This behavior clearly shows the influence of solar radiation on the development of different types of biofouling. In particular, the presence of light favors the development of photosynthetic fouling.

Following the biofouling cleaning procedure of the Al–Mg surfaces (Figure 4), different Al–Mg surface modifications were observed on each side of exposure, light and dark. The light side (Figure 4a) shows a nonuniform appearance, with the presence of more dull zones than others. This behavior becomes less evident for longer immersion time, revealing the Al–Mg surface at the end of 6 months of immersion a more uniform appearance. No localized attacks of the Al alloy were observed until 6 months of exposure. In contrast, the dark side exhibits a more pronounced alteration on its surface. This is evident not only through the change in surface color and the traces of calcareous organisms (e.g., tube worms), more evident with immersion time, but also due to the visible pitting attack of the AA5083 surface from 2 months of exposure (Figure 4, indicated by the red circle). For both cases, light and dark sides, the aluminum alloy surface showed after 6 months of immersion a cracked surface morphology, which was easily observed for the dark side samples (Figure 4, higher magnification).

### 3.2 Scanning Electron Microscopy (SEM) with Energy Dispersive X-ray Analysis (EDX)

SEM/EDX characterization of AA5083 samples with different immersion times in seawater, light and dark sides, and after biofouling cleaning was performed on surfaces and cross-sections. The results are shown in Figures 5–9.

**Figure 5:**
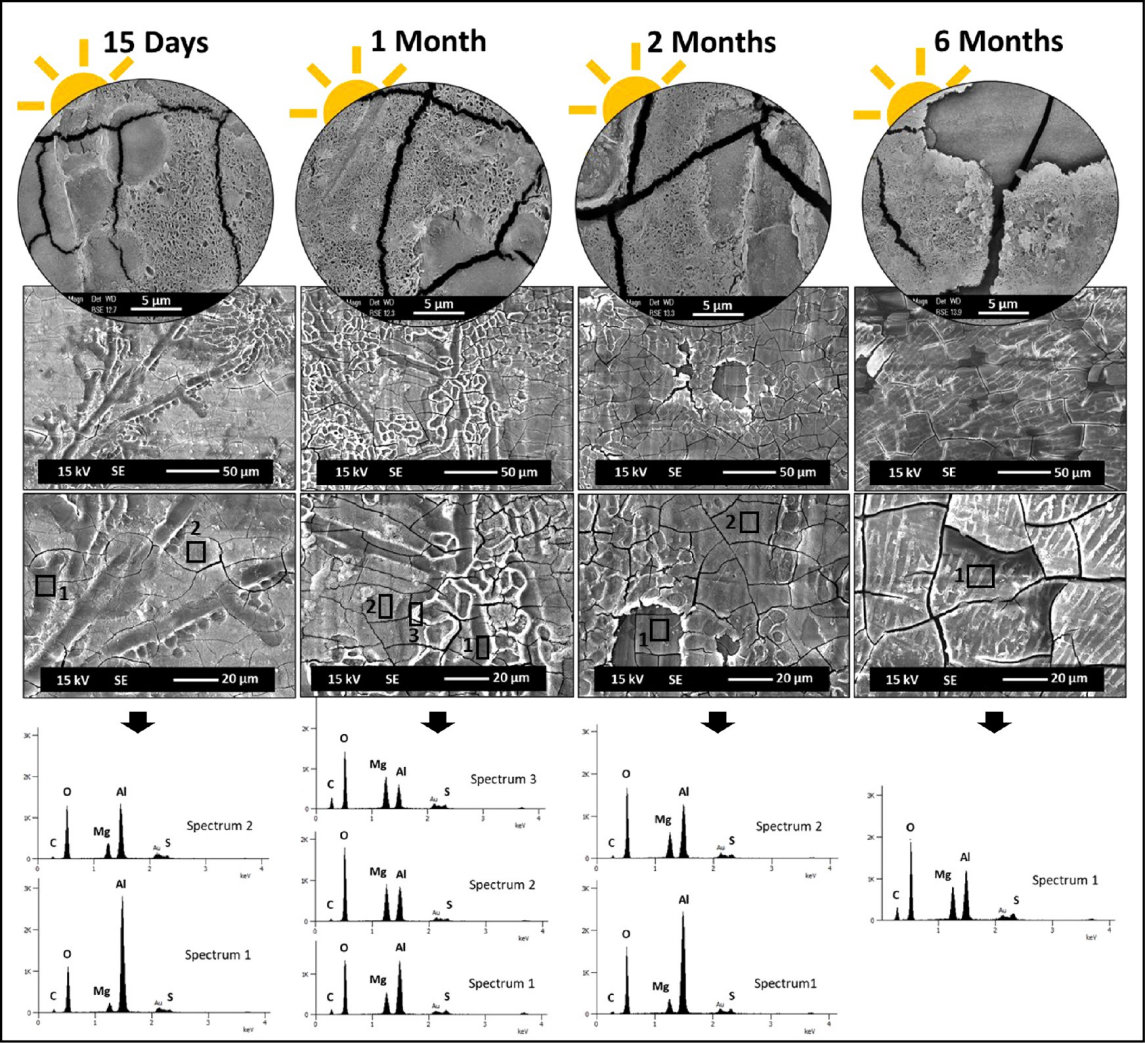
SEM/EDX surface characterization of Al–Mg samples immersed for 15 days and 1, 2, and 6 months in seawater (after biofouling removal). Light side of exposure.

Figure 5 shows the Al–Mg surfaces exposed on the light side, which reveal surface morphology modifications linked to the evolution of colonization by microorganisms during the immersion time. The drawing shape of the physionomy of microorganisms previously present on the Al–Mg surfaces is more pronounced on the samples immersed for a short period of time, 15 days and 1 months, respectively. A cracked surface is observed (as already noticed by digital microscopy observation) with no evidence of localized corrosion, up to the 6 months of immersion. At higher magnification, a needle-shaped morphology is visible with a heterogeneous surface distribution, revealing an increase in density (less porous) with the immersion time (Figure 7a).

The elemental chemical analysis carried out by EDX (spectra in Figure 5) identified oxygen (O), aluminum (Al), and magnesium (Mg) as the main chemical elements present. Sulfur (S) and carbon (C) were also identified, although with lower intensities. The EDX analysis carried out on the Al–Mg surface with a needle-shaped morphology revealed an enrichment in Mg compared to the zones without this type of morphology.

In contrast, the Al–Mg surfaces exposed on the dark side during the immersion test, although revealing a cracked surface (Figure 6), showed a significantly less marked needle-shaped morphology (Figure 7b) and visible pitting corrosion from 2 months of immersion (Figure 6). The EDX analysis performed near the corrosion pits revealed areas with the metallic substrate exposed and the presence of few aluminum corrosion products (spectra in Figure 6). In the zones without localized corrosion, the surface showed no considerable heterogeneity and no Mg surface enrichment was detected, in contrast to Al–Mg surfaces exposed to the light side.

**Figure 6:**
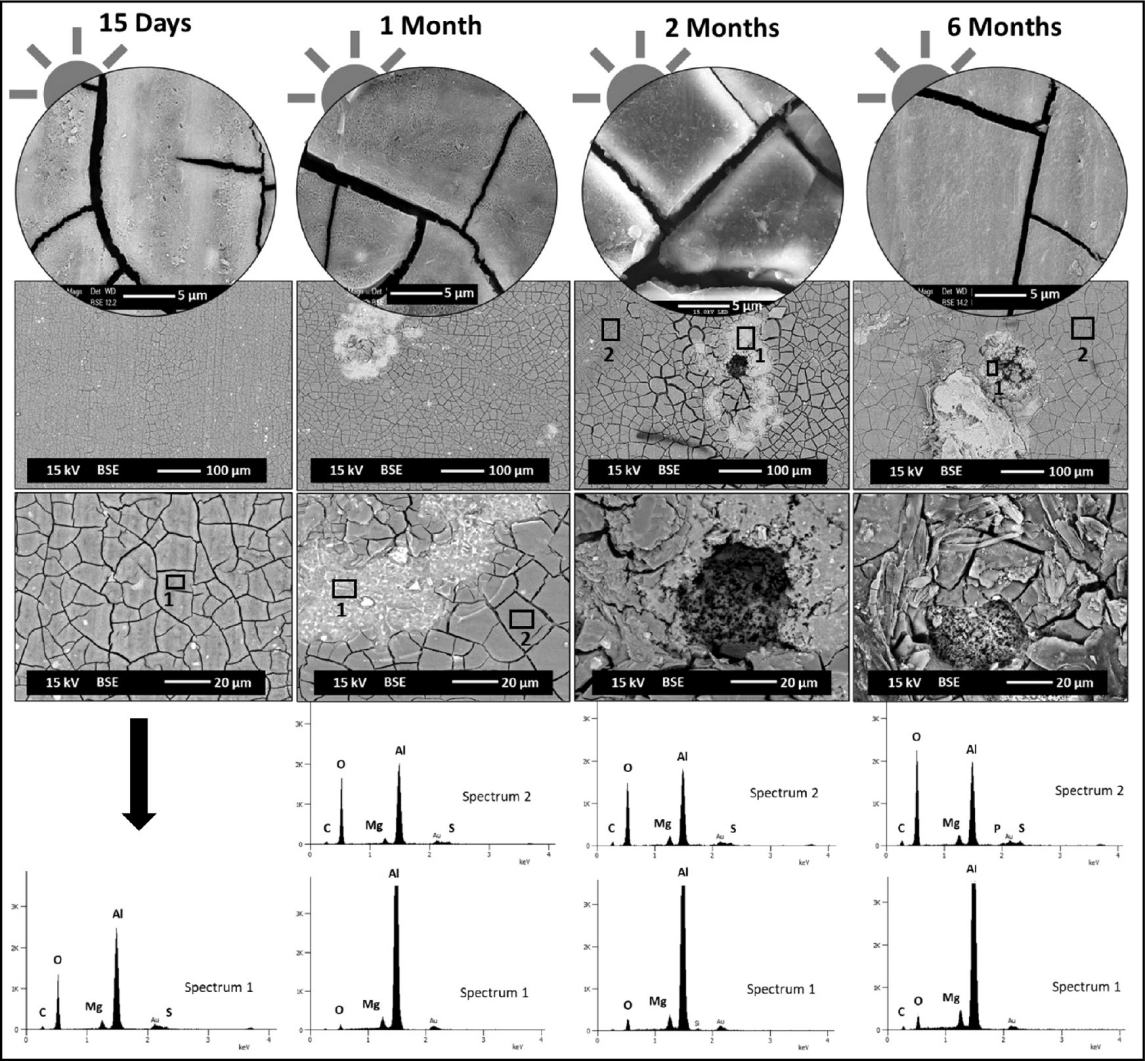
SEM/EDX surface characterization of Al–Mg samples immersed for 15 days and 1, 2, and 6 months in seawater (after biofouling removal). Dark side of exposure.

**Figure 7:**
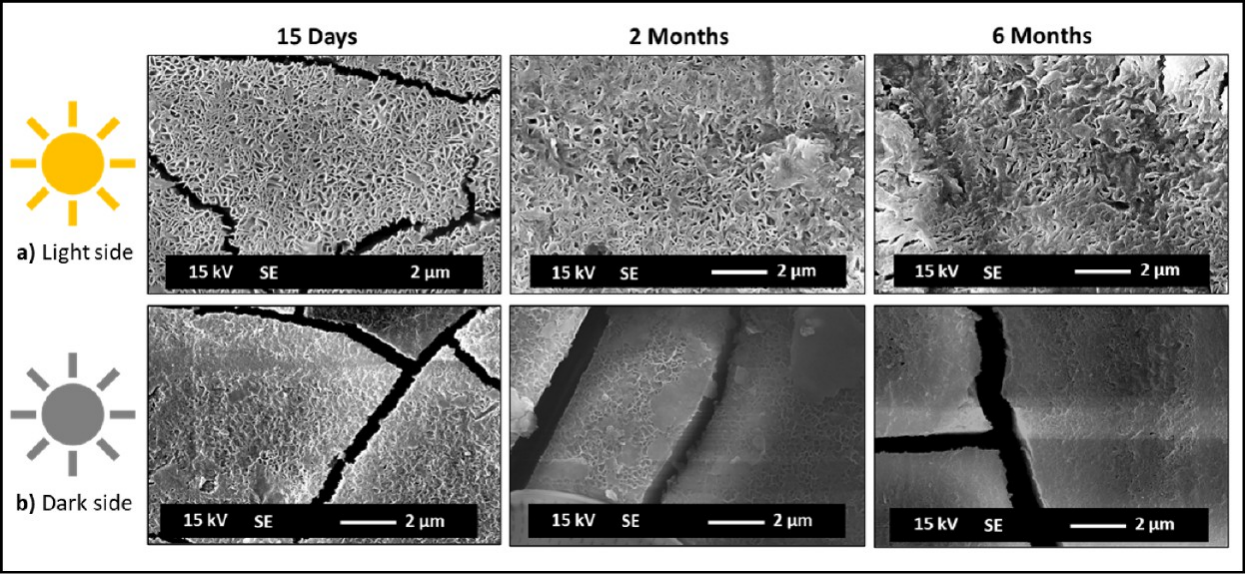
SEM surface observation of Al–Mg samples immersed for 15 days, 2 months, and 6 months. Light (a) and dark (b) sides of exposure.

Regarding the cross-section characterization of the Al–Mg surfaces exposed to the light side, the observations confirmed the absence of a localized attack of the metal substrate during the seawater immersion test. Figure 8 summarizes the SEM/EDX results for the aluminum alloy immersed up to 6 months. A dual-layer structure is observed on the aluminum alloy, with a nonuniform distribution over the whole substrate. An inner layer, with a compact appearance exhibiting an average thickness of 3.5 μm, and an outer layer, with an average thickness of about 0.7 μm, nonuniformly distributed and showing a lamellar appearance. The analysis performed by EDX revealed the qualitative difference in elemental chemical composition between the two layers, inner and outer, confirming an Mg-rich outer layer (Figure 8—spectra 3 and 4) comparatively with the inner layer, richer in Al (Figure 8—spectra 1 and 2). The presence of calcium (Ca) was also identified in the inner layer, although with a significantly lower presence compared to the other chemical elements detected.

**Figure 8:**
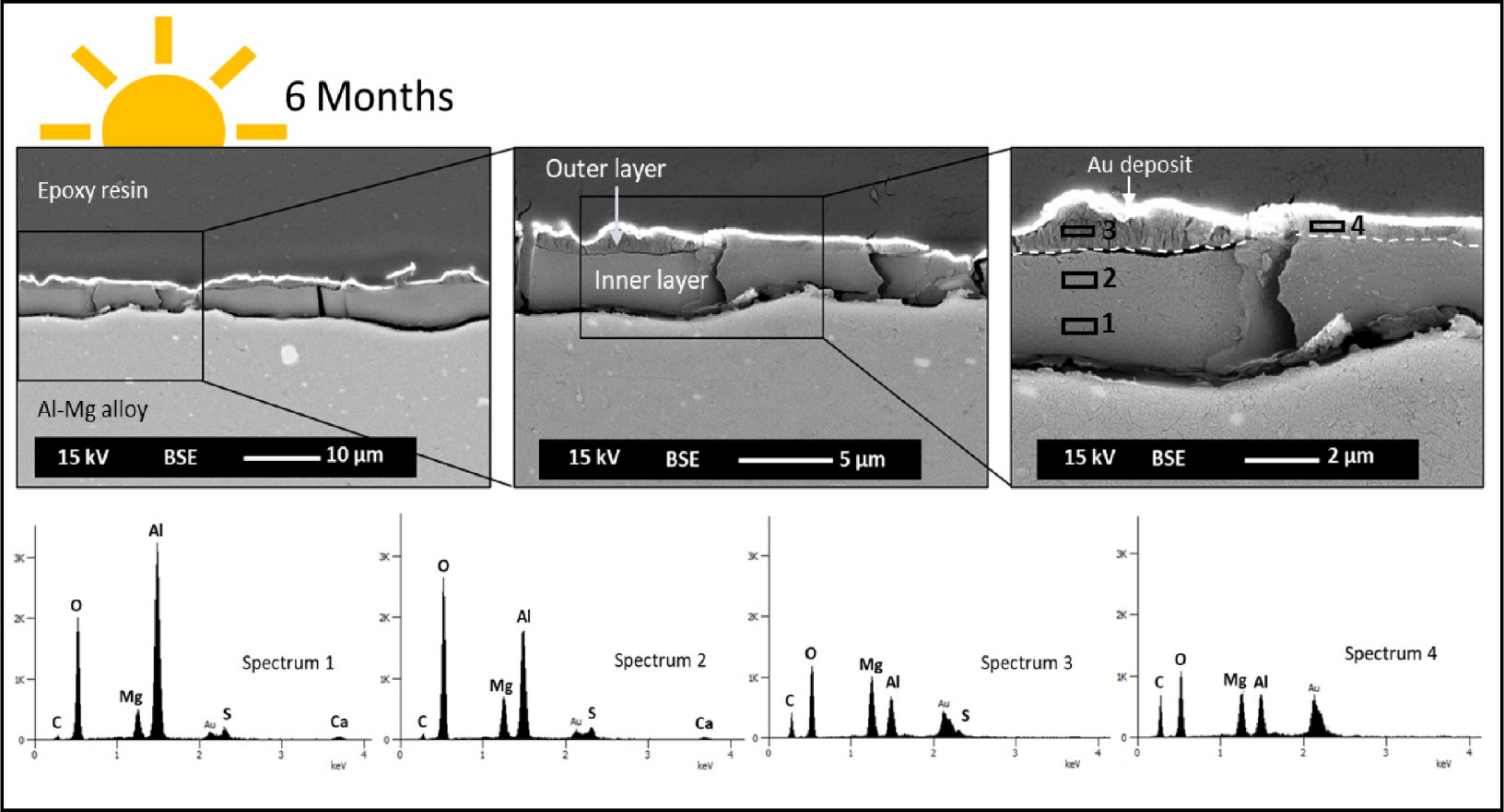
SEM/EDX cross-section characterization of Al–Mg samples after 6 months of immersion. Light side of exposure.

The cross-section characterization of the Al–Mg surfaces exposed on the dark side reveals a localized attack of the alloy from two months of immersion (Figure 9). In zones without localized corrosion of the aluminum alloy, a single layer is observed, heterogeneously distributed and with an average thickness that increases with immersion time. For the longest immersion test (6 months), the average layer thickness was 6.3 μm. From 2 months of immersion, disintegration of the layer is observed, as well as loss of adhesion and, where the layer was absent, a localized attack of the Al–Mg alloy.

**Figure 9:**
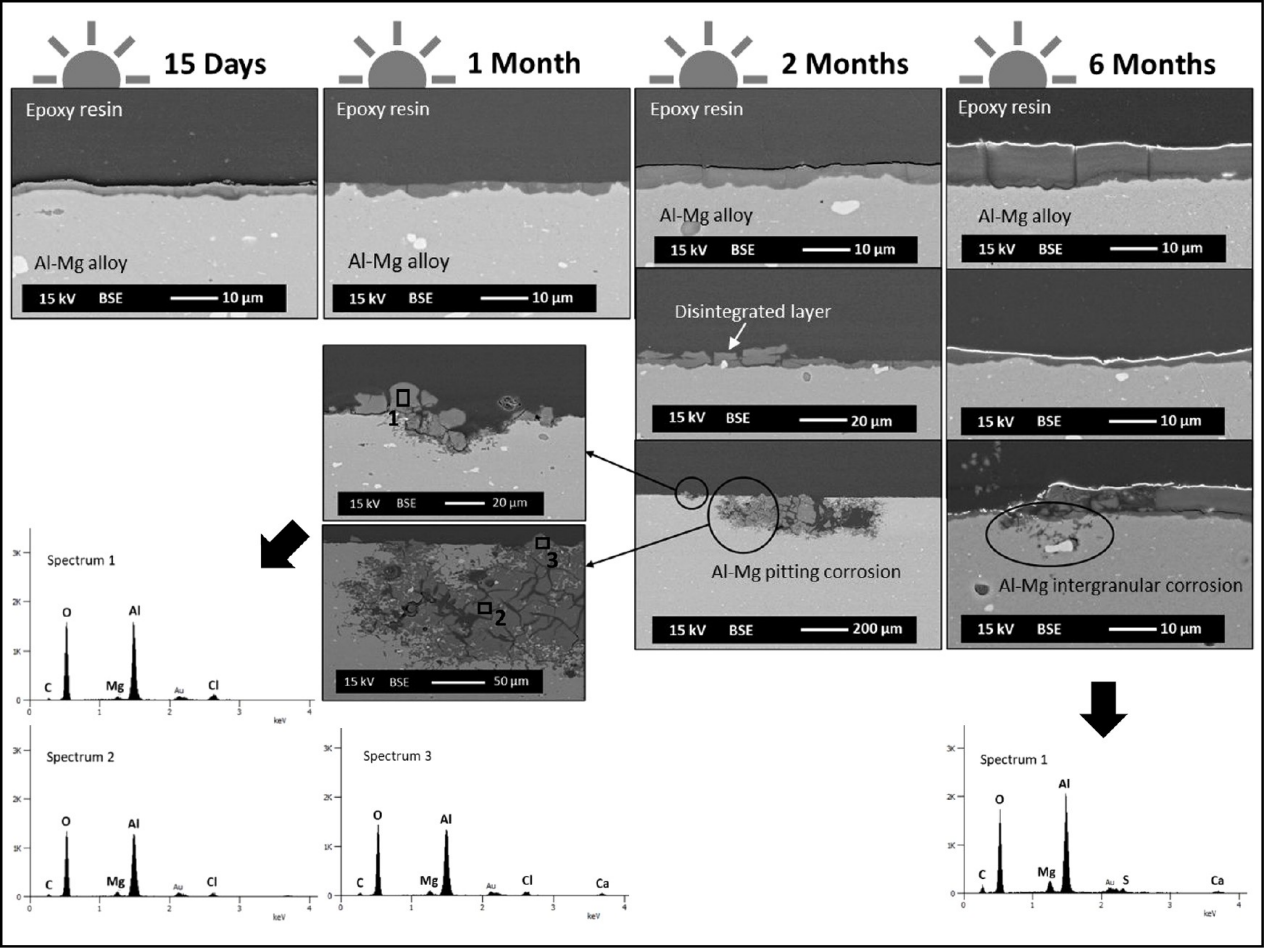
SEM/EDX cross-section characterization of Al–Mg samples after 15 days and 1, 2, and 6 months of immersion. Dark side of exposure.

The corrosion products analyzed by EDX in the attacked zones revealed the presence of chloride (Cl). At the zones without localized corrosion of the aluminum alloy, i.e., in the layer adjacent to the metal substrate, the EDX results are similar to those obtained for the inner layer observed on the light side, an aluminum-rich layer.

In summary, over the immersion period, the surface modifications of the Al–Mg alloy are influenced by the side of exposure and the type of fouling formed on the surface, i.e., predominantly photosynthetic or not. On the light side, the Al–Mg surface, previously covered with different types of algae, did not show any localized attack. The surface modification resulted in the formation of a dual-layer structure, with an inner layer rich in Al and an outer layer rich in Mg. For the dark side of exposure, where the fouling species initially removed from the Al–Mg surface were mostly nonphotosynthetic (calcareous type), localized corrosion was detected from 2 months of immersion. In the nonattacked zones, a single layer, rich in Al, adjacent to the substrate was observed (similar to the inner layer of the Al–Mg samples exposed to the light side), showing a significant thickness increase with immersion time.

Figure 10 shows the evolution of the average thickness of the layer formed adjacent to the Al–Mg alloy surface over the immersion period. Up to 1 month of immersion, even though different presences of fouling and surface modifications have been already observed, the response of the Al–Mg surface shows similar reactivity, forming an Al-rich layer with an average thickness of around 2 μm on both sides of exposure (light and dark). After this period, the alloy surface reveals a clearly different behavior, with the dark side showing a significant increase in the average layer thickness compared to the light side. At 2 months of immersion, an average thickness of 1.8 μm for the light side and almost the double for the dark side, 3.5 μm, is observed. This result highlights different corrosion kinetics between the light and dark sides, with the dark side revealing a faster one. After 6 months of immersion, the Al–Mg surfaces exposed to the light side showed no localized corrosion and an average thickness of the Al-rich layer of 3.5 μm, almost twice lower than that observed for the dark side (6.3 μm).

**Figure 10:**
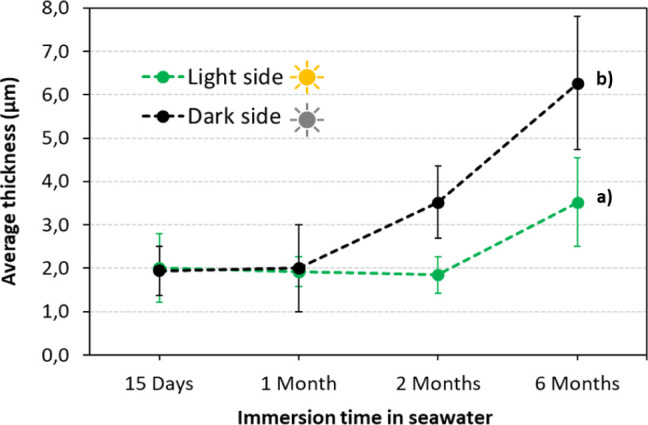
Evolution of the average thickness of the layer formed adjacent to the Al–Mg alloy surface, light (a) and dark (b) sides of exposure, during the seawater immersion test.

Figure 11 compares the Al–Mg surfaces exposed to the light and dark sides after 6 months of immersion (cross-section observations), revealing the clear difference between both samples regarding the thickness of the layer adjacent to the surface of the Al–Mg alloy. Additionally, for the Al–Mg surfaces exposed to the light side, the dual-layer structure, with the presence of the Mg-rich outer layer, is visible.

**Figure 11:**
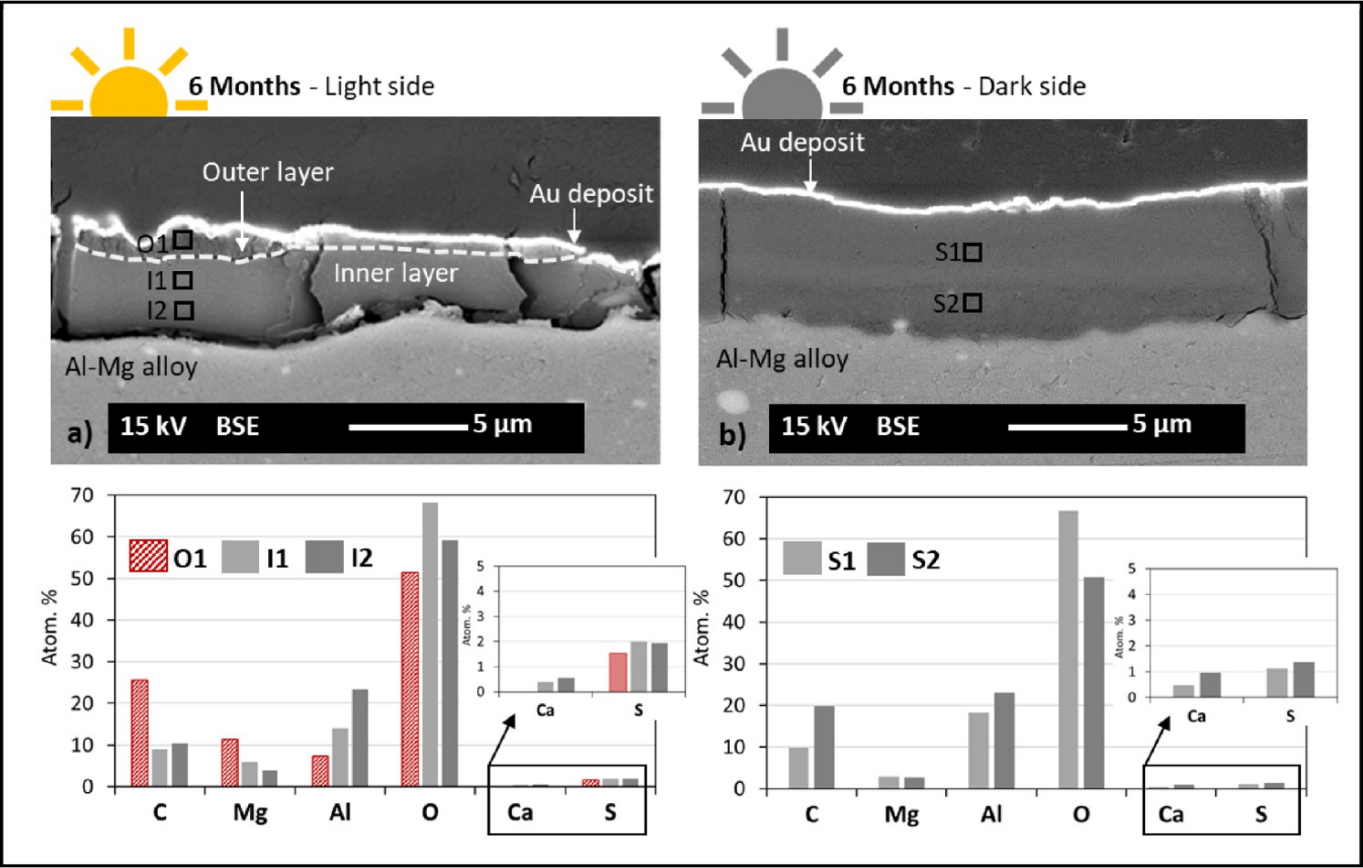
Local comparison of the light and dark sides of Al–Mg samples after 6 months of immersion. Cross-sectional SEM micrographs and graphics with atomic % of the chemical elements identified by EDX for (a) light side (dual structure: outer layer (O1) plus inner layer (I1/I2)) and (b) dark side (single layer structure (S1/S2)).

The results of the EDX analysis on both samples, light and dark side, presented in the form of a bar graphic (atomic % vs chemical elements) (Figure 11), aimed only a qualitative interpretation to allow an easier visualization of the different elemental chemical composition of the different layers formed during the immersion test. The adjacent layer to the Al–Mg substrate shows a similar elemental composition for both samples, with variations in the chemical elements identified throughout the thickness of the layer (with the presence of aluminum (Al) and oxygen (O) prevailing). It is worth noting that in the case of calcium (Ca) and sulfur (S), which are significantly less present compared to the other elements identified, the Al–Mg samples exposed on the light side show a higher presence of sulfur compared to the samples exposed on the dark side, while the Al–Mg samples exposed on the dark side show a higher presence of calcium (Ca) compared to the samples exposed on the light side. The outer layer, present only on the Al–Mg surfaces exposed to the light side, is clearly richer in Mg than the inner layer.

### 3.3 Grazing Incidence X-ray Diffraction (GIXRD)

The presence of crystalline compounds was investigated by GIXRD analysis after for 6 months of immersion.

The diffraction pattern of the Al–Mg alloy immersed on the light side of exposure, even though it showed peaks with low intensity, allowed the identification of a layered double hydroxide (LDH)-type compound (Figure 12). The reference to both compounds, magnesium aluminum hydroxide hydrate, Mg_6_Al_2_(OH)_18_.4.5­(H_2_O) (JCDD Pdf 00-035-0965), and meixnerite, Mg_6_Al_2_(OH)_18_·4H_2_O (JCDD Pdf 00-038-0478), is due to the fact that, with the obtained diffraction pattern, compounds with 4 or 4.5 water molecules are hardly distinguished. Comparing the results obtained for the dark side, no LDH-type compound was identified. The absence of detectable crystalline calcareous compounds on the surface may be associated with a low crystalline state of the present phases. In each case, light and dark side, given that the samples studied come from immersion in field conditions, some difficulties were expected using this technique. As observed by SEM, the Al–Mg surface modification are not uniform or homogeneous, and the roughness is high, which affects the GIXRD analysis.

**Figure 12:**
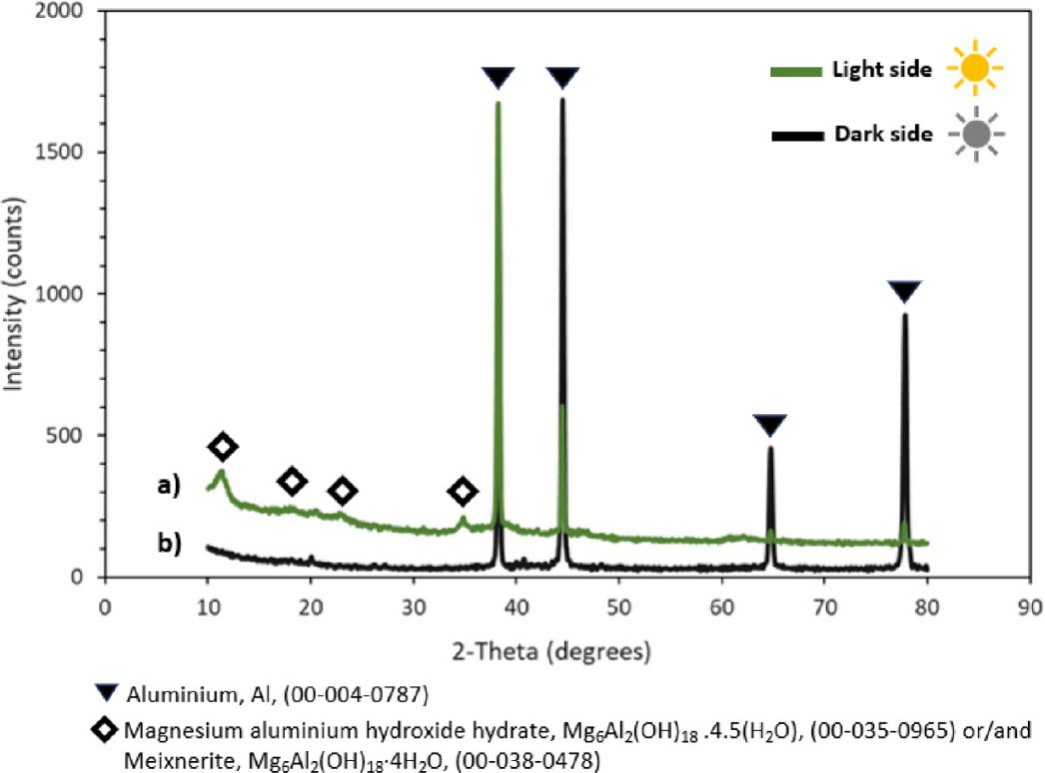
GIXRD patterns of Al–Mg samples after 6 months of immersion. Light (a) and dark (b) sides of exposure.

### 3.4 μ-Raman Spectroscopy (μ-RS)

In order to complement the results obtained by GIXRD, Figure 13 shows the results obtained by μ-RS analysis, performed on the Al–Mg surfaces after 6 months of immersion, light and dark side of exposure.

**Figure 13:**
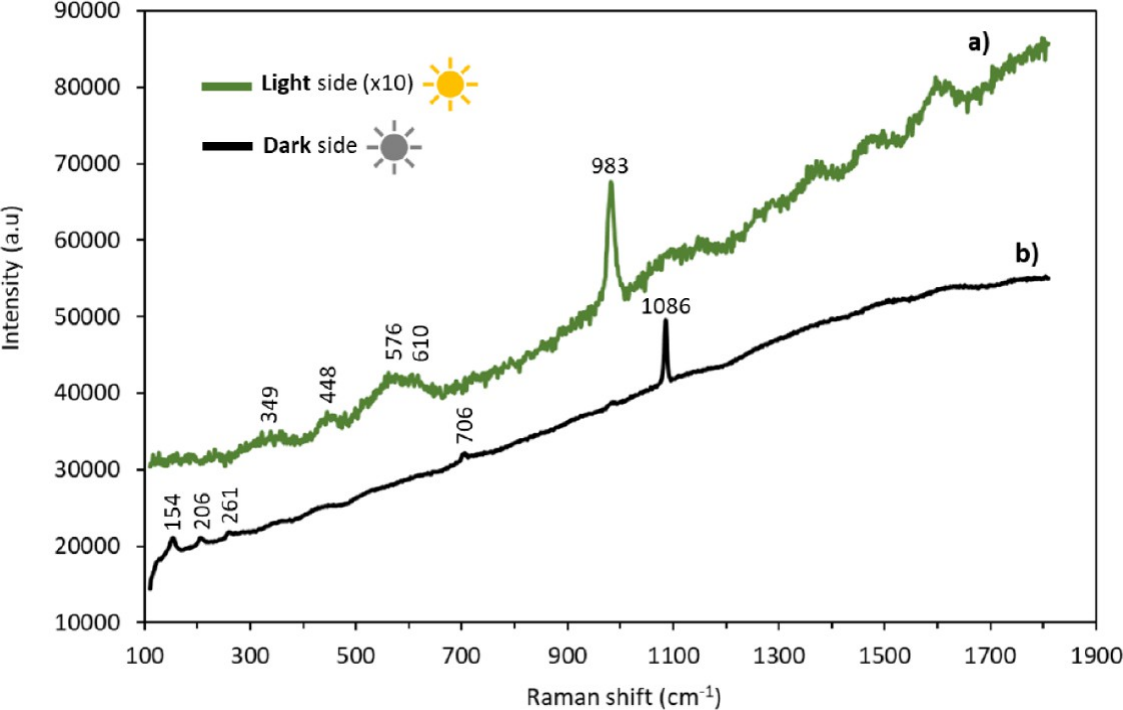
Raman spectra obtained on Al–Mg samples after 6 months of immersion. Exposure of light (a) and dark (b) sides.

For the light side, a typical SO_4_^2–^ vibrational band is identified in the μ-RS spectra around 982 cm^–1^, corresponding to the symmetrical stretching vibration (ν_1_) of the SO_4_^2–^ group. Other vibrational modes of the group are also visible, even if they are of lower intensity. These include the ν_2_ and ν_4_ modes around 450 and 610 cm^–1^, respectively, corresponding to deformation modes (symmetrical and asymmetrical).^42^

A spectral signature including the vibrational bands of the SO_4_^2–^ groups is also visible at low wave numbers between 200 and 600 cm^–1^ (Raman vibration bands at 349, 448, and 576 cm^–1^), which can be associated with magnesium and aluminum hydroxy sulfates, such as the Mg–Al hydrotalcite-like compounds^43^ (e.g., Mg_6–*x*_Al_2+__*x*_(OH)_16_(SO_4_).*y*H_2_O).

The oscillations indicated between 1150 and 1800 cm^–1^ are not Raman vibrations but a response of the interference filter linked to intense fluorescence emitted by the sample.

In the case of the Al–Mg surface exposed to the dark side, the presence of aragonite (CaCO_3_) was identified. μ-RS spectra show a band corresponding to the carbonate symmetrical stretching vibration at 1085 cm^–1^ (ν_1_) and other vibrational modes are also visible, with lower intensity.^44^ These include the ν_2_ and ν_4_ modes at 154 and 206 cm^–1^, respectively, corresponding to angular deformation. At 706 cm^–1^ (ν_1_), the peak is related to the chain deformation mode. No peak associated with sulfate ion was identified.

### 3.5 X-ray Photoelectron Spectroscopy (XPS)

The highly surface sensitive XPS technique allows us to obtain information from the top of the surface to a few nanometers in depth and to perform quantitative analysis (atomic concentrations of elements present), which is particularly useful in this case, to assess the chemical composition changes occurring on the Al–Mg alloy surface after the different immersion times in seawater (for light and dark sides of exposure).

#### 3.5.1 XPS In-Depth Analysis of Surface Layers

Argon ions (Ar^+^) sputtering was performed in order to investigate the in-depth composition of the surface layers over about 100 nm, which corresponds to a total of three etching cycles with XPS analysis.

Figure 14 shows the XPS spectra of the Mg 2p, Al 2p, Mg 2s, Al 2s, and S 2p core levels obtained at the end of the third etching cycle for each of the Al–Mg surfaces for light (Figure 14a) and dark (Figure 14b) sides of exposure. For both exposure conditions, the peaks at 74.3 and 119.2 eV are associated with Al (III) and peaks at 50.4 and 90 eV are associated with Mg (II), indicating that the surface is mainly composed of Al and Mg oxides and hydroxides.^45^ A comparison between the XPS spectra of Figure 14a,b shows a higher intensity of the magnesium peak for the light side than for the dark side. A S 2p peak at high binding energy, 168.3 eV, is assigned to sulfate species.^45^ For long immersion times, longer than 2 months, the intensity of this peak is much lower for the dark side than for the light side.

**Figure 14:**
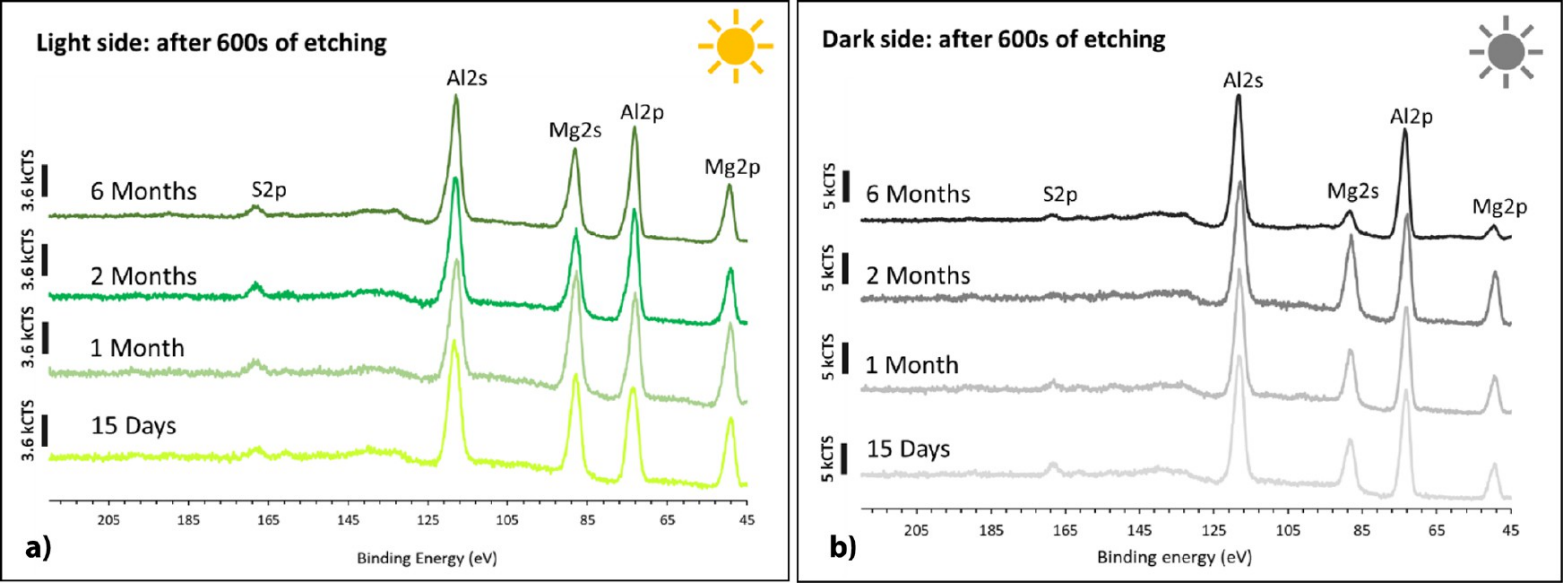
XPS spectra obtained on Al–Mg surfaces immersed for 15 days and 1, 2, and 6 months, light (a) and dark (b) sides of exposure, after 600 s of etching (approximate 100 nm depth, 0.2 nm s^–1^).

Curve-fitting of high-resolution O 1s spectra, recorded after 600 s of etching (approximately 100 nm depth), for all the samples, light and dark sides of exposure, is presented in Figure 15. A comparison between the spectra shows two common components at binding energies of 531 and 532.8 eV, associated with the presence of oxygen in the form of oxides and hydroxides.^45^ However, in the case of the Al–Mg surfaces exposed to the light side, all the spectra obtained revealed the presence of one more component at a binding energy of 535.3 eV assigned to the presence of water,^45^ whose intensity significantly increases with immersion time. For the samples exposed on the dark side, only a residual presence of water was detected for the Al–Mg surfaces after 2 months of immersion.

**Figure 15:**
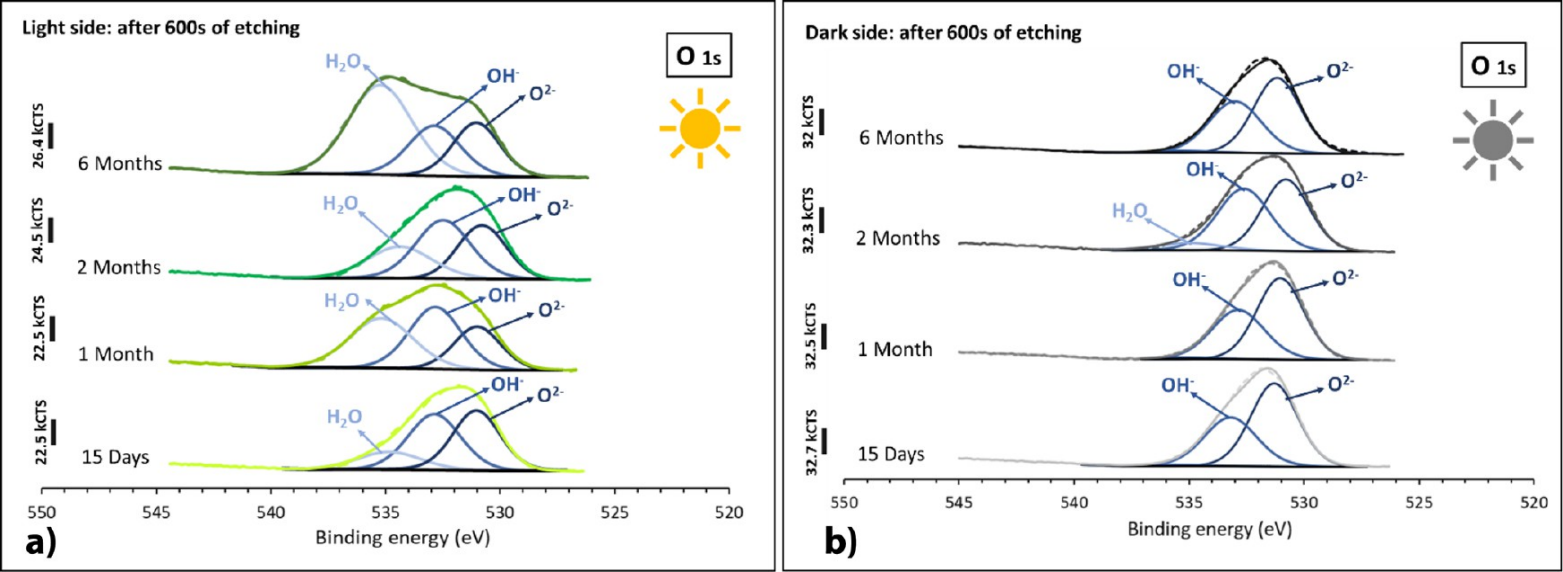
XPS O 1s spectra obtained on Al–Mg surfaces immersed for 15 days and 1, 2, and 6 months, light (a) and dark (b) sides of exposure, after 600 s of etching (depth approximately 100 nm, 0.2 nm s^–1^).

Figure 16 (atomic concentrations (%) vs etching time (s)) shows the changes in composition during the depth profile analysis from the surface to a depth of about 560 nm (2800 s of sputtering) for the 6-months immersed samples, light and dark sides.

**Figure 16:**
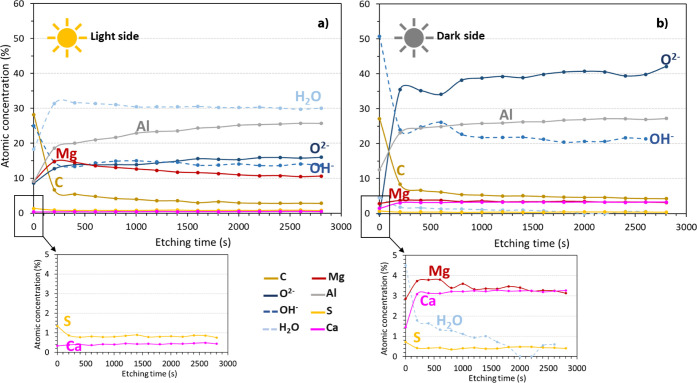
XPS depth profiles results for the Al–Mg samples immersed during 6 months in seawater, light (a) and dark (b) sides of exposure, after 14 etching cycles of 200 s (approximately 560 nm depth, 0.2 nm s^–1^).

As the outer layer of the dual structure observed on the Al–Mg surface exposed on the light side has an average thickness of 0.7 μm (SEM characterization), it can be assumed that the XPS analysis has been performed inside this outer layer. In the case of the Al–Mg surface exposed on the dark side, the analysis concerned the external part of the single layer, which was 6.3 μm-thick.

For both exposure conditions, light and dark sides, the atomic concentration of C drops immediately after the first sputtering cycle (*t* = 200 s) and then remains almost constant throughout the sputtering cycles. The high atomic concentration of C detected for t = 0 s can be associated with the presence of extracellular polymeric substances (EPS) linked to biological activity (more homogeneously distributed on the light side) but also to residual presence on the extreme surface of calcareous structures produced by marine organisms (micro and macro), more significant on the dark side.

At t = 200 s (after the first sputtering), it is possible to observe a significant presence of H_2_O on the Al–Mg surface exposed to the light side, with an atomic concentration (%) 17.5 times higher than that detected on the Al–Mg surface exposed to the dark side. The difference remains high throughout the profile.

Moreover, for magnesium (Mg) profiles, a higher atomic concentration is observed in the samples exposed to light. The highest value is detected at t = 200 s, approximately 15% Mg (at %), which is 4 times higher than for the dark side.

The presence of sulfur (S), although significantly lower (atomic concentration value, for t = 200 s, below 1%) is 2 times higher for surfaces on the light side than for the dark side.

The presence of oxygen (O) is very differently distributed for the Al–Mg surfaces exposed at the light or dark sides: for the light side, the main component was water, whereas the O^2–^ and OH^–^ forms are much more pronounced for the dark side, with an atomic concentration (at %) 3 and 2 times higher, respectively, than for the light side.

Regarding calcium (Ca), although with an atomic concentration of less than 5%, its presence on the Al–Mg surfaces exposed to the dark side is 8 times higher than that for the light side. This higher Ca value on the dark side is certainly influenced by the remaining calcareous structures on the alloy surface, as observed after the cleaning of the biofouling.

These results indicate that the outer layer of the dual structure observed on the Al–Mg surface exposed to light during 6 months of immersion is significantly hydrated and richer in magnesium, while the external part of the single layer, observed on the Al–Mg surface exposed to dark during 6 months of immersion, is richer in oxides and hydroxides, with a higher prevalence of oxides.

#### 3.5.2 XPS Characterization of Organic Compounds Present on Al–Mg Surfaces after 6 Months of Immersion

The presence and distribution of extracellular polymeric substances (EPS), a complex mixture of biopolymers resulting from marine biological activity (such as polysaccharides, proteins, and other organic compounds), can be influenced by the type of microfouling (bacteria, diatoms, and microalgae) and macrofouling (algae, tubeworms, barnacles, and mussels) that produce them. In this context, Figure 17 shows XPS data on the organic matter present on the Al–Mg extreme surface after 6 months of immersion, for the light and dark sides, as a result of the biological activity.

**Figure 17:**
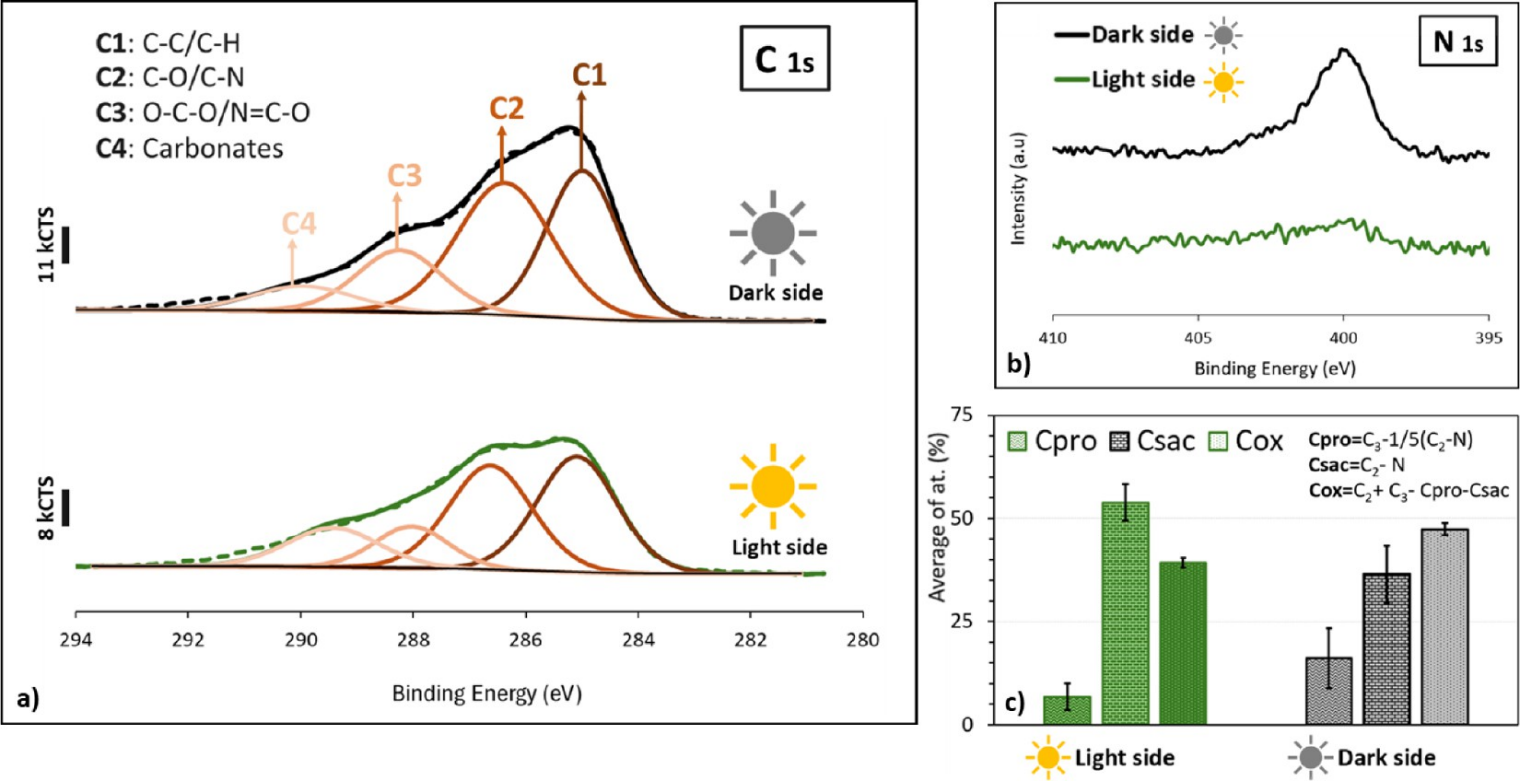
(a) XPS spectra of carbon C 1s core level obtained on Al–Mg surfaces immersed for 6 months (light and dark side). (b) XPS spectra of nitrogen N 1s core level obtained on Al–Mg surfaces after 6 months of immersion. (c) Average of the atomic percentage of proteins (Cpro), polysaccharides (Csac), and oxygenated species (Cox) obtained on Al–Mg surfaces exposed to the light and dark sides for 6 months immersion.

The fitting of the C 1s spectra for the Al–Mg surfaces after 6 months of immersion (Figure 17), for the light and dark sides, reveals, for both cases, four components (C1–C4) as described in Section 2.3.5.2.

For the N 1s spectrum (Figure 17), a marked difference is observed between the Al–Mg surfaces immersed with and without solar exposure. The surfaces exposed to the dark side show one component associated with the amide/amine groups (400.1 eV), significantly more intense than observed for the light side.

The quantitative analysis presented in Figure 17c is based on the data obtained from the C 1s and N 1s spectra recorded at four distinct points per surface analyzed (light and dark sides). According to the standard deviation (Figure 17c), the distribution of EPS on the Al–Mg surface appeared to be more homogeneous on the light side than on the dark side. The results revealed the presence of more polysaccharides on the light side (54%) compared to the dark side (37%) and twice the amount of protein on the dark side (16%) compared to that on the light side (7%). The amount of oxygenated species was higher on the dark side (47%) than on the light side (39%).

Polysaccharides, proteins, and oxygen species are constituents of EPS, and their presence and distribution are influenced by the type of microfouling (bacteria, diatoms, and microalgae) and macrofouling (algae, tubeworms, barnacles, and mussels) that produce them.

### 3.6 Summary Discussion

Figure 18 shows a scheme summarizing the modifications of the alloy surface over the period of 6 months of immersion in seawater for the light and dark sides of exposure. This is based on the surface and interface characterization results reported in this work (additional data in Table S1).

**Figure 18:**
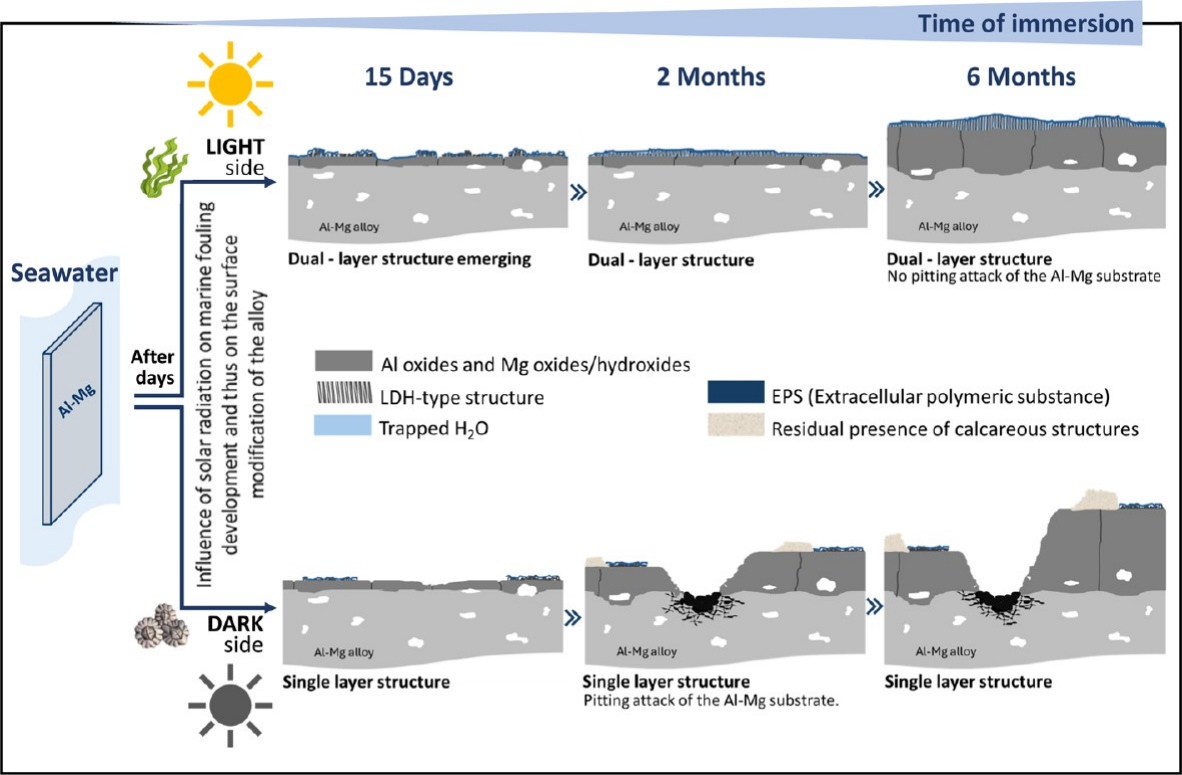
Schematic summary of the Al–Mg surface modifications with the time of immersion in seawater for the light and dark sides of exposure. Cross-section representation.

After 15 days of immersion, macroscopic observation of the Al–Mg alloy surfaces revealed differences in the initial development of macrofouling settlement (Figure 1) depending on solar exposure. The light side presents the first appearance of algae, and the dark side presents the presence of tubeworms formation. SEM observations combined with EDX and XPS analysis performed on both sides allowed us to verify differences in surface modifications according to the type of fouling developed (photosynthetic or not). Although in both cases, light and dark sides, a layer with a similar elemental chemical composition and average thickness has formed on the substrate, resulting from the process of anodic dissolution of the Al matrix, the extreme surfaces showed a different behavior. On the light side, a lamellar-shaped morphology was observed, which, by EDX and XPS analysis, was found to be rich in magnesium and hydrated, with the presence of EPS. A cross-sectional SEM observation, using an alternative method to classic metallographic preparation, allowed us to confirm that this surface modification resulted in the formation of a second layer, which has grown from the rich Al oxide/hydroxide layer adjacent to the substrate (Figure S2). This behavior was not observed on the dark side. In this latter case, there was no surface enrichment in magnesium (absence of a lamellar surface morphology) and no detectable presence of water.

The results for 1 month of immersion showed a continuation of the behavior observed after 15 days of testing, i.e., the difference between the light and the dark sides of exposure.

After 2 months of immersion, macro- and microscale characterization confirms even more pronounced differences. The light side showed an evolution of the colonization of the surface, essentially by the so-called soft-fouling, in contrast to the dark side, where hard fouling is mostly present. In both cases, this behavior continues to be translated in different Al–Mg surface modifications. On the Al–Mg exposed to the light side, the formation of a dual-layer structure is confirmed, in which the inner Al rich layer showed no significant thickness evolution, unlike the outer layer (rich in Mg) whose thickness increased and also was more hydrated. Despite the heterogeneous distribution of this thin outer layer, μ-RS analysis allowed identification of a Mg–Al hydrotalcite-like compound (Mg_6–*x*_Al2+_*x*_(OH)_16_(SO_4_).*y*H_2_O) (Figure S3). This result suggests that the observed previous growth of the Mg-rich layer, when favorable conditions of divalent and trivalent ion concentration and alkaline pH are maintained, enables the ongoing process of precipitation and the formation of a Mg–Al LDH-type compound.

Oppositely, on the dark side, no presence of the Mg-rich lamellar structure (no outer layer) was observed, and the thickness of the Al-rich layer increased (almost double the inner layer for the light side, Figure 10), revealing a different corrosion kinetic. Additionally, some zones of the surface already showed localized corrosion.

At this point, it is important to highlight previously published results related to the study of the impact of marine biological activity on the Al–Mg alloy during 2 months of immersion in seawater,^35^ in which ToF-SIMS analysis clearly showed that the Mg-rich outer layer developed in biotic-light immersion had a barrier effect, revealing a decrease in the diffusion of Cl^–^ ions, in contrast to the dark side of exposure where the presence of the Mg-rich outer layer was not observed. Thus, it can be considered that the outer layer formed on the light side played a role on the inhibition corrosion process of the Al–Mg alloy: limiting the uniform corrosion (thin inner layer) and preventing the localized corrosion by restricting Cl^–^ ions diffusion. On the contrary, on the dark side, the presence of high chloride amount, mainly in the areas where the attack shows to be more intense, largely explained the pitting corrosion.^35^

After 6 months of immersion, the behavior is in agreement with the previous findings and proves to be consolidate. The surface and interface characterization of the tested samples allowed us to confirm (i) the evolution of the average thickness of the Al-rich layer adjacent to the substrate, which continues to be approximately 2 times superior on the dark side than on the light side. This behavior confirms the different corrosion kinetics of the Al–Mg alloy according to the side of exposure; (ii) the evolution of the Mg-rich outer layer observed on the Al–Mg alloy surface exposed on the light side, although heterogeneously distributed, revealed at this stage an increase in the average thickness (Figure 8), making it possible to identify by GIXRD and μ-RS analysis the presence of magnesium aluminum hydroxide hydrate and magnesium aluminum hydroxy sulfate compounds. These results confirm that the outer layer rich in Mg, at this stage, forms an LDH-type structure. In addition, the results of the XPS analysis also confirmed a high hydration of the Mg-rich outer layer as well as a relatively homogeneous presence of EPS on its extreme surface. In contrast to the Al–Mg surface exposed on the dark side, where no Mg-rich outer layer is observed, no presence of water was detected, and the presence of EPS proved to be more heterogeneous. (iii) The corrosion-inhibition role played by the Mg-rich outer layer, i.e., the highly hydrated LDH-type structure, is confirmed after 6 months of immersion, with the Al–Mg surface exposed to the light side showing no pitting corrosion and a lower anodic dissolution. This behavior contrasts with the dark side, where a more accelerated anodic dissolution of the Al matrix and pitting corrosion of the Al–Mg alloy can be seen as early as 2 months of immersion.

Summarizing, at this stage, it is clear that the outer layer composed of an LDH-type structure plays a role in the corrosion process of the alloy. This raises the following questions: (i) Why, during immersion of the Al–Mg alloy in seawater, does the formation of the outer layer (dual-layer structure) occur only on the Al–Mg surfaces exposed to the light side and (ii) Is the corrosion inhibition effect of the outer layer only due to the presence of an LDH type structure?

#### 3.6.1 Light vs Dark for LDH Growth

During the immersion process of the Al–Mg alloy in seawater, solar radiation proved to have an impact on the type of colonization that is developed on the metal surface (photosynthetic or nonphotosynthetic), which in turn was revealed to have a decisive influence on the surface modification that occurred on the alloy, leading to a corrosion-inhibiting effect or not. XPS analysis performed for both sides of exposure, light and dark (see Figure 17), revealed differences in the chemical composition of the EPS, according to the type of fouling. On the light side, the EPS produced by the photosynthetic soft fouling were enriched in polysaccharides consistent with the presence of marine algae on the alloy surface, while on the dark side, the EPS were characterized by a higher content of proteins, linked to the presence of nonphotosynthetic hard fouling, such as tubeworms and barnacles.

Marine algae (unicellular or colonial microalgae and multicellular marine organisms) are an inexhaustible source of various polysaccharides, including the group of sulfated polysaccharides (SPs) such as carrageenans in red algae (Rhodophyta), alginates and fucoidans in brown algae (Phaeophyceae), and ulvans in green algae (Chlorophyta). The sulfated polysaccharides exhibit a notable hydrogel-forming ability due to their hydrophilic nature and the presence of sulfate groups, which attract water molecules.^46–57^ Consistent with this characteristic, the XPS depth profile of the Al–Mg surface immersed for 6 months exposed on the light side revealed a significantly higher content of H_2_O compared to the dark side (Figure 16). When sulfated polysaccharides come into contact with water, the sulfate groups can form hydrogen bonds with water molecules, leading to water absorption and retention. This property is crucial in imparting a gel-like consistency to the EPS. Additionally, the presence of divalent cations in seawater, such as Ca^2+^ and Mg^2+^, may facilitate multiple cross-linkages between different polysaccharide chains. These cross-linkages may enhance the overall stability and consistency of the EPS matrix, creating favorable conditions for ion adsorption necessary for mineral structure formation. In the present particular case, the Mg–Al–OH/Mg–Al LDH-like structure formation was promoted. Indeed, seawater acting as a source of divalent cations such as Mg^2+^ combined with the presence of sulfated polysaccharides on the surface of the Al–Mg alloy, where oxidation–reduction reactions generate trivalent cations (Al^3+^) and lead to alkaline pH conditions (as a result of corrosion phenomena), provides suitable conditions for the formation of LDH-type structures. To the best of our knowledge, it was the first time that the in situ LDH growth was shown in a real environment. Usually, the in situ LDH growth on metal substrates is achieved under well-controlled laboratory conditions mainly based on electrodeposition and coprecipitation methods.^58–67^

In contrast, the XPS measurements performed on the Al–Mg surfaces exposed on the dark side indicated a lower concentration of sulfate (Figure 14) and polysaccharides and a significantly higher presence of nitrogen compared to the light side (Figure 17b,c). Hard-fouling organisms, such as barnacles and tubeworms, are primarily known for producing EPS rich in proteins and polysaccharides to attach firmly to surfaces. However, these polysaccharides are typically not sulfated ones. Despite the lack of sulfate groups, they may still have some water-absorbing capacity depending on their chemical structure, but it is generally lower than that of sulfated polysaccharides. Additionally, the higher presence of nitrogen on the Al–Mg surface can be linked to a more significant development of organisms, which, in the absence of light, use nitrogen fixation as a source of energy. This increase in the availability of nitrogen compounds may have an influence on the surface modifications by competing for ions, typically involved in LDH formation, as OH^–^ ions and metal cations (e.g., Mg^2+^ and Al^3+^). This certainly contributes to the inhibition process of LDH growth on the dark side.

#### 3.6.2 Corrosion Inhibition Effect of the Biomineralized Layer (“Outer Layer”)

After 6 months of immersion in seawater, no pitting was observed on the surface exposed to the light side and a lower uniform corrosion kinetics was found compared to the surface exposed on the dark side. This behavior was associated with the formation of a double-layer structure in which the outer layer, composed of a highly hydrated LDH-type structure covered by EPS, proved to play a role in the corrosion inhibition process of the alloy (Figure 19). As previously mentioned, the presence of EPS, in particular sulfated polysaccharides (SPs), would promote the in situ growth of LDH on the Al–Mg surface by improving the adsorption of ions from seawater. In addition, the higher water absorption capacity of these polysaccharides, which increases their ability to form hydrogels,^68,69^ would allow to fill the space between the LDH flakes, leading to a reduced diffusion rate of aggressive ions to the metal surface and increasing the physical barrier effect.

**Figure 19:**
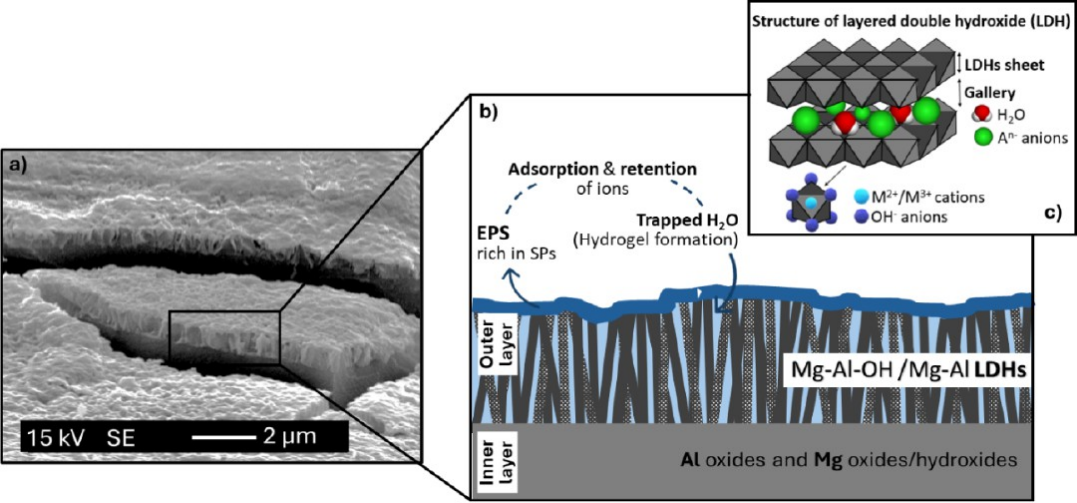
(a) SEM observation of Al–Mg surface modification after 6 months of immersion and light side of exposure (prepared by bending process), (b) cross-section illustration of the biomineralized layer (“outer layer”) present on the dual layer structure formed on the Al–Mg surface after 6 months of immersion and light side of exposure, and (c) schematic representation of a typical structure of LDH.

In this context, the corrosion-inhibiting behavior of the biomineralized layer formed is explained as functioning as a hybrid inorganic–organic protective system, in which the LDH layer acts as the first ″defensive line” and the presence of an impregnated hydrogel and EPS on the Al–Mg surface plays a decisive role in enhancing the anticorrosion properties of the overall system.

## 4 Conclusion

The Al–Mg alloy behavior study conducted from the first 15 days up to 6 months of immersion in seawater revealed the significant impact of solar radiation on fouling development and, consequently, on the modification of the alloy surfaces, highlighting the distinct corrosion kinetics observed between the light and dark sides of exposure. The presence of photosynthetic soft fouling on the light side, dominated by marine algae, undergoes the formation of an Mg-rich outer layer that is significantly hydrated, which evolves into a Mg–Al LDH-type structure. The results presented in this paper are the first observation of in situ LDH growth on an Al–Mg alloy in a real marine environment.

This layer acts as a corrosion-inhibiting barrier limiting uniform and localized corrosion. In contrast, the dark side, dominated by nonphotosynthetic hard fouling organisms like barnacles and tubeworms, does not develop this protective layer, showing a more accelerated anodic dissolution and pitting corrosion.

This study demonstrates, for the first time, that EPS enriched in sulfated polysaccharides produced by marine algae can influence the in situ growth of a Mg–Al LDH compound on the Al–Mg surface immersed in seawater and enhance the physical barrier effect improving the corrosion resistance of the alloy.

## Supplementary Material



## Data Availability

The raw/processed data required to reproduce these findings cannot be shared at this time as the data also form part of an ongoing study.

## Notes

Experimental setup for the in situ marine immersion test (CNRS-IAS Genoa Marine Station); SEM observation of Al–Mg samples after 15 days of immersion (light exposure); Raman spectra of Al–Mg samples after 2 months of seawater immersion (light exposure); summary data of Al–Mg surface modifications according to immersion time and exposure conditions (PDF)
