# Supplementary material for: Exploring Marine Biomineralization on the Al–Mg Alloy as a Natural Process for In Situ LDH Growth to Improve Corrosion Resistance
Source: ACS Appl Mater Interfaces. 2025 Jan 30;17(6):10038–54. doi: 10.1021/acsami.4c17532 (PMC12818716; doi:10.1021/acsami.4c17532)
Supplement: Supplementary file 1 [file am4c17532_si_001.pdf]

Supporting Information

Exploring marine biomineralization on Al-Mg alloy as a natural process for in situ LDH growth to improve corrosion resistance.

Maria João F. Marques[1,2*], Dimitri Mercier[3], Antoine Seyeux[3], Sandrine Zanna[3*], Christophe Tenailleau[4], Benjamin Duployer[4], Marc Jeannin[5], Philippe Marcus[3] and Régine Basséguy[1*]

[1] Laboratoire de Génie Chimique, Université de Toulouse, CNRS, INPT, UPS, Toulouse, 31432, France

[2] Laboratório de Materiais e Revestimentos, Laboratório Nacional de Energia e Geologia (LNEG), Lisboa, 1649-038, Portugal

[3] Institut de Recherche de Chimie Paris, Research Group Physical Chemistry of Surfaces, Chimie ParisTech-CNRS, PSL Research University, Paris, 75005, France

[4] Centre Interuniversitaire de Recherche et d'Ingénierie des Matériaux, Université de Toulouse 3, CNRS, UPS, Toulouse, 31062, France

[5] Laboratoire des Sciences de l´Ingénieur pour l´Environnement, CNRS, Université de La Rochelle, La Rochelle, 17042, France

*Corresponding authors: mjoao.marques@lneg.pt; regine.basseguy@toulouse-inp.fr; sandrine.zanna@chimieparistech.psl.eu

Supplementary Figures

2. Materials and methods

2.2. In situ marine immersion test

The marine immersion test was carried out on the platform available at the CNR-IAS Genoa Experimental Marine Station (GEMS).

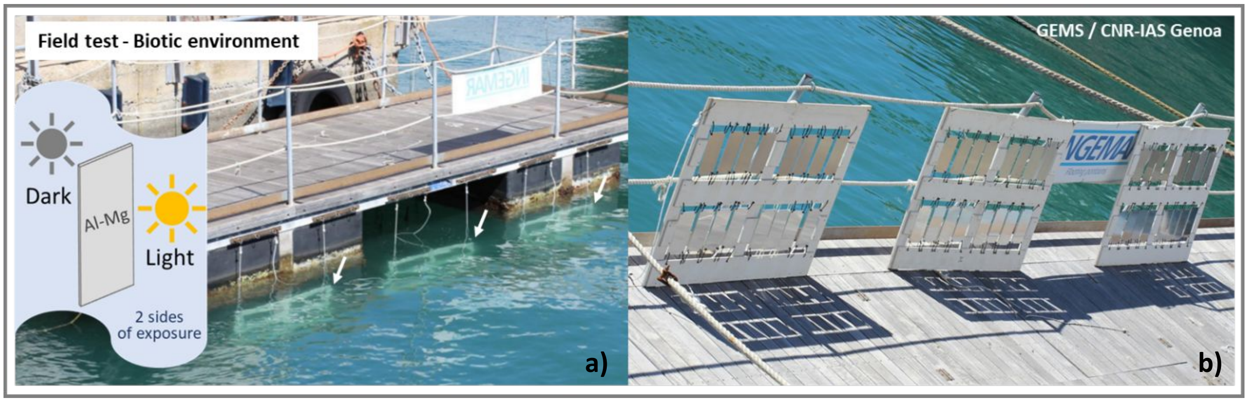

Fig.S1 - Immersion of the Al-Mg samples in natural seawater at the CNRS – IAS Genoa experimental marine station (GEMS): (a) Immersion position of the frames (indicated by the arrows) with two different exposure sides, light and dark, and (b) Frames before seawater immersion.

4. Discussion

A cross-sectional SEM observation, using an alternative method to classic metallographic preparation, allowed us to confirm that this surface modification resulted in the formation of a second layer, which has grown from the rich Al oxides/hydroxides layer adjacent to the substrate.

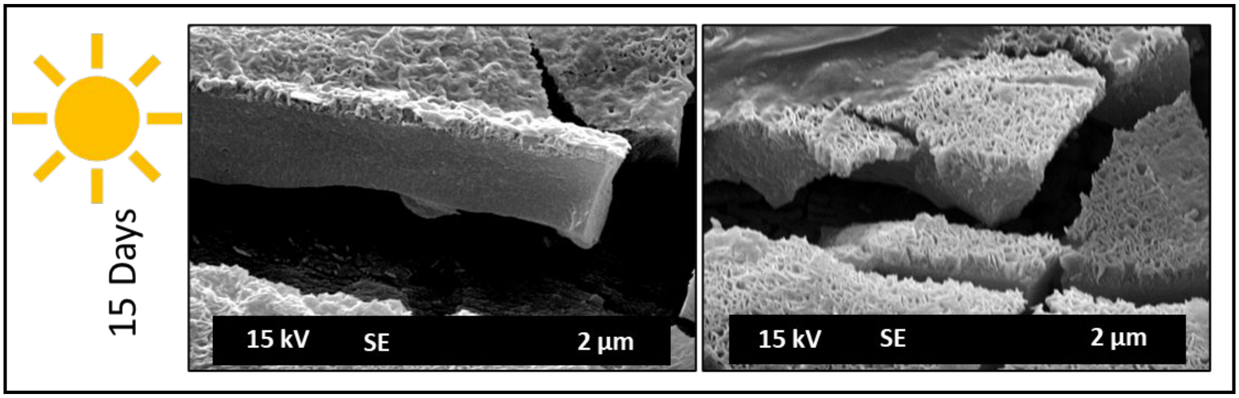

Fig.S2 - SEM observation of Al-Mg samples immersed for 15 days, light side of exposure. Surface characterization after a bending process.

4. Discussion

On the Al-Mg exposed to the light side, the formation of a dual-layer structure is confirmed, in which the inner Al rich layer showed no significant thickness evolution, unlike the outer layer (rich in Mg) whose thickness increased and also was more hydrated. Despite the heterogeneous distribution of this thin outer layer, μ-RS analysis allowed to identify a Mg-Al hydrotalcite-like compound (Mg_6-$xAl_{2+x}(OH)_{16}(SO_4).yH_2O$).

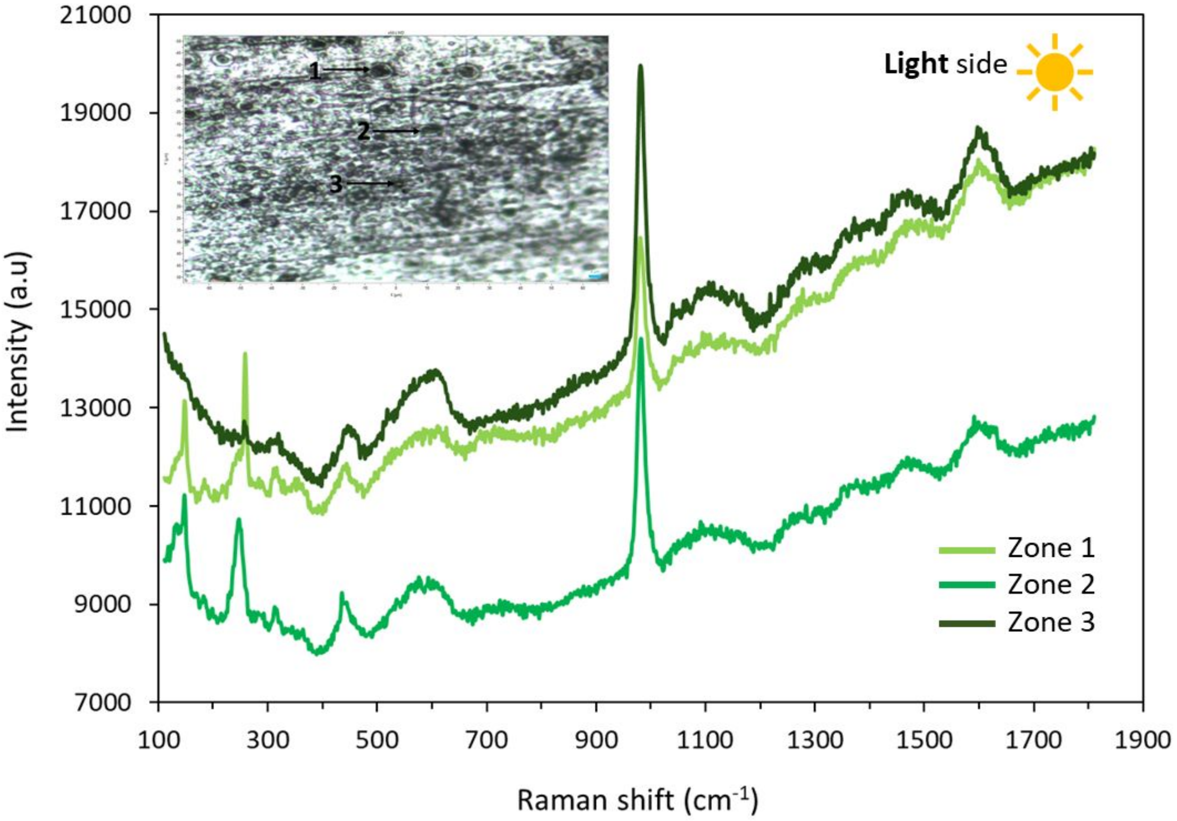

- Typical $\mathbf{SO_4^{2-}}$ vibrational band is identified in the μ-RS spectra around 980 cm^{-1}, corresponding to the symmetrical stretching vibration (V_1) of the SO_4^{2-} group.
- Zones 1 and 2, show a spectral signature which include the vibrational bands of the **SO_4^{2-} groups**, at low wave numbers between 200 and 600 cm^{-1}, that can be associated to **magnesium and aluminium hydroxy sulphates**, such as the **Mg-Al hydrotalcite-like compounds** (example, Mg_6-$xAl_{2+x}(OH)_{16}(SO_4).yH_2O$).
- The oscillations indicated between 1150 and 1800 cm^{-1} are linked to intense fluorescence emitted by the sample.

Fig.S3 - Raman spectra obtained in different zones of the Al-Mg samples after 2 months of immersion. Light side of exposure.

4. Discussion

Table S1 summarises the modifications of the alloy surface over the period of 6 months of immersion in seawater, for the light and dark sides of exposure. This is based on the surface and interface characterization results reported in this work.

Table S1 - Summary of the Al-Mg surface modifications with the time of immersion in seawater, for the light and dark sides of exposure.

**Exposure Conditions**	**Time of Seawater Immersion**		
	**15 Days**	**2 Months**	**6 Months**
**LIGHT** side<br>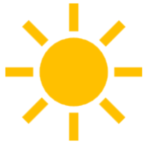	**Dual - layer structure emerging**<br>(i) Outer layer < 0.5 µm (average)<br>• Organic matter associated to EPS.<br>• H_2O presence.<br>• Mg surface enrichment. (heterogeneously distributed)<br>• Sulphate on the surface.<br>(ii) Inner layer ≈ 2 µm (average)<br>• Al rich layer with presence of Ca. (Al matrix dissolution, anodic process)	**Dual - layer structure**<br>(i) Outer layer ≈ 0.5 µm (average)<br>• Organic matter associated to EPS covering the extreme surface.<br>• Hydrated outer layer.<br>• Mg enrichment.<br>• Sulphate on the surface.<br>• Presence of $Mg_6\text{-}xAl_{2+x}(OH)_{16}(SO_4).yH_2O$ (Mg-Al hydrotalcite-like compound).<br>(ii) Inner layer ≈ 1.8 µm (average)<br>• No pitting attack of the Al-Mg substrate.	**Dual - layer structure**<br>(i) Outer layer > 0.5 µm (average)<br>• Organic matter associated to EPS covering the extreme surface.<br>• Highly hydrated outer layer.<br>• Mg enrichment. Presence $Mg_6Al_2(OH)_{18}.4.5(H_2O)$ or/and $Mg_6Al_2(OH)_{18}{\cdot}4H_2O$ (Mg-Al-OH type LDH)<br>• Sulphate on the surface. Presence of $Mg_6\text{-}xAl_{2+x}(OH)_{16}(SO_4).yH_2O$ (Mg-Al hydrotalcite-like compound).<br>(ii) Inner layer ≈ 3.5 µm (average)<br>• No pitting attack of the Al-Mg substrate.
**DARK** side<br>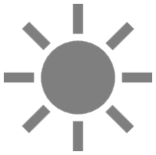	**Single layer structure**<br>(i) Extreme surface<br>• Some organic matter associated to EPS.<br>• No H_2O detected.<br>• Lower Mg surface enrichment.<br>• Sulphate on the surface.<br>(ii) Layer ≈ 2 µm (average)<br>• Al rich layer with presence of Ca. (Al matrix dissolution, anodic process)	**Single layer structure**<br>(i) Extreme surface<br>• Some organic matter associated to EPS and residual presence of calcareous structures linked to the previous presence of hard fouling.<br>• Lower Mg surface enrichment.<br>• Low amount of H_2O.<br>• Lower sulphate presence.<br>(ii) Layer ≈ 3.5 µm (average)<br>• Localized corrosion. Pitting attack of the Al-Mg substrate.	**Single layer structure**<br>(i) Extreme surface<br>• Some organic matter associated to EPS and more residual presence of calcareous structures linked to the previous presence of hard fouling.<br>• No H_2O detected.<br>• Significantly lower Mg surface enrichement.<br>• Lower sulphate presence.<br>(ii) Layer ≈ 6.3 µm (average)<br>• Localized corrosion. Pitting attack of the Al-Mg substrate.
